# A TBR1-K228E Mutation Induces *Tbr1* Upregulation, Altered Cortical Distribution of Interneurons, Increased Inhibitory Synaptic Transmission, and Autistic-Like Behavioral Deficits in Mice

**DOI:** 10.3389/fnmol.2019.00241

**Published:** 2019-10-09

**Authors:** Chaehyun Yook, Kyungdeok Kim, Doyoun Kim, Hyojin Kang, Sun-Gyun Kim, Eunjoon Kim, Soo Young Kim

**Affiliations:** ^1^Department of Biological Sciences, Korea Advanced Institute for Science and Technology (KAIST), Daejeon, South Korea; ^2^Center for Synaptic Brain Dysfunctions, Institute for Basic Science (IBS), Daejeon, South Korea; ^3^Division of National Supercomputing, Korea Institute of Science and Technology Information (KISTI), Daejeon, South Korea; ^4^College of Pharmacy, Yeongnam University, Gyeongsan, South Korea

**Keywords:** autism spectrum disorder, Tbr1, transcription factor, cortical development, GABAergic neurons, synaptic transmission, social and repetitive behavior

## Abstract

Mutations in *Tbr1*, a high-confidence ASD (autism spectrum disorder)-risk gene encoding the transcriptional regulator TBR1, have been shown to induce diverse ASD-related molecular, synaptic, neuronal, and behavioral dysfunctions in mice. However, whether *Tbr1* mutations derived from autistic individuals cause similar dysfunctions in mice remains unclear. Here we generated and characterized mice carrying the TBR1-K228E *de novo* mutation identified in human ASD and identified various ASD-related phenotypes. In heterozygous mice carrying this mutation (*Tbr1*^+/K228E^ mice), levels of the TBR1-K228E protein, which is unable to bind target DNA, were strongly increased. RNA-Seq analysis of the *Tbr1*^+/K228E^ embryonic brain indicated significant changes in the expression of genes associated with neurons, astrocytes, ribosomes, neuronal synapses, and ASD risk. The *Tbr1*^+/K228E^ neocortex also displayed an abnormal distribution of parvalbumin-positive interneurons, with a lower density in superficial layers but a higher density in deep layers. These changes were associated with an increase in inhibitory synaptic transmission in layer 6 pyramidal neurons that was resistant to compensation by network activity. Behaviorally, *Tbr1*^+/K228E^ mice showed decreased social interaction, increased self-grooming, and modestly increased anxiety-like behaviors. These results suggest that the human heterozygous TBR1-K228E mutation induces ASD-related transcriptomic, protein, neuronal, synaptic, and behavioral dysfunctions in mice.

## Introduction

Autism spectrum disorders (ASD) are brain developmental disorders characterized by social deficits and repetitive behaviors. A large number of genetic mutations associated with ASD have been identified (Rosti et al., 2014; De Rubeis and Buxbaum, 2015; de la Torre-Ubieta et al., 2016; McDermott et al., 2018), and potential mechanisms underlying ASD have been identified (Südhof, 2008; Bourgeron, 2009, 2015; Kleijer et al., 2014; Barak and Feng, 2016; Hulbert and Jiang, 2016; Golden et al., 2018). However, knock-in animals carrying heterozygous ASD-risk mutations from individuals with ASD have been less thoroughly investigated.

TBR1, a neuron-specific T-box transcription factor, regulates the regional and laminar identity of neocortical regions, including layer 6, in the developing brain (Bulfone et al., 1995, 1998; Dwyer and O’Leary, 2001; Hevner et al., 2001, 2002; Englund et al., 2005; Kolk et al., 2006; Bayatti et al., 2008; Bedogni et al., 2010; Han et al., 2011; McKenna et al., 2011; Cánovas et al., 2015; Marinaro et al., 2017; Elsen et al., 2018; Liu et al., 2018). Neuronal activation upregulates the expression of *Tbr1*, which together with CASK, a synaptic PDZ protein that can translocate into the nucleus, and CINAP, a nucleosome assembly protein, form a complex that regulates the expression of target proteins, including reelin and the GluN2B subunit of N-methyl-D-aspartate receptor (NMDAR), involved in the regulation of brain development and function (Hsueh et al., 2000; Wang G. S. et al., 2004; Wang T. F. et al., 2004; Chuang et al., 2014; Huang et al., 2014; Klionsky et al., 2016). More recently, TBR1 was found to directly interact with FOXP2 (Sakai et al., 2011; Deriziotis et al., 2014), a transcription factor associated with brain development and speech, as well as language impairments (Enard et al., 2002; Fisher and Scharff, 2009; Enard, 2011; Tsui et al., 2013).

Reflecting this critical role of TBR1 in brain and cortical development, *TBR1* has been strongly associated with brain disorders, including ASD and intellectual disability (Neale et al., 2012; O’Roak et al., 2012, 2014; Traylor et al., 2012; De Rubeis et al., 2014; Deriziotis et al., 2014; Hamdan et al., 2014; Palumbo et al., 2014; Chuang et al., 2015; Sanders et al., 2015; Bowling et al., 2017; Geisheker et al., 2017; McDermott et al., 2018; Vegas et al., 2018); among the many other genes on the SFARI (Simons Foundation Autism Research Initiative) list, it is considered a category 1 high-confidence ASD-risk gene (Abrahams et al., 2013). In addition, TBR1 has been shown to regulate the expression of various ASD-risk genes (Chuang et al., 2014, 2015; Huang et al., 2014; Notwell et al., 2016; Fazel Darbandi et al., 2018), likely as part of a large network of genes involved in ASD.

More recently, a multitude of neurobiological mechanisms that may underlie TBR1-dependent development of ASD have been reported in studies using *Tbr1*-mutant mice (Huang and Hsueh, 2015). Specifically, a *Tbr1* haploinsufficiency has been shown to diminish amygdalar projections and induce autism-like behaviors (including reduced social interaction, cognitive inflexibility and impaired associative memory) that can be corrected by direct and indirect activation of NMDARs (Huang et al., 2014; Lee et al., 2015). In addition, layer 6-specific deletion of TBR1 leads to the loss of excitatory and inhibitory synapses in layer 6 pyramidal neurons, and anxiety-like and aggressive behaviors (Fazel Darbandi et al., 2018). A *Tbr1* haploinsufficiency also induces impairments in olfactory discrimination (but not olfactory sensation) that are improved by NMDAR activation (Huang et al., 2019). Although these results provide significant insights into how TBR1 dysfunctions lead to ASD, whether and how *TBR1* mutations identified in humans lead to ASD remains unclear.

Here, we generated and characterized a knock-in mouse line carrying the TBR1-K228E mutation identified in a 7-year-old male with ASD (O’Roak et al., 2012). This mutation, localized to the TBR1 protein T-box domain involved in DNA binding and protein-protein interaction, has been shown to disrupt the interaction between TBR1 and FOXP2 (Deriziotis et al., 2014), without affecting TBR1 nuclear localization, homodimerization, CASK interaction, or transcriptional-repression activity. These experiments, performed in HEK293 cells, suggested however that a portion of TBR1-K228E protein targeted to the nucleus form abnormal aggregates in heterologous cells (Deriziotis et al., 2014). Although these findings provide important clues as to the potential pathophysiology of the TBR1-K228E mutation, whether mice carrying a heterozygous TBR1-K228E mutation (*Tbr1^+/K228E^* mice) display ASD-related behaviors and related molecular and cellular abnormalities remain unknown. We report here that *Tbr1*^+/K228E^ mice show a multitude of ASD-related phenotypes at protein, transcriptomic, cellular, synaptic and behavioral levels, findings that differ in certain aspects from previous results obtained in mice carrying a null allele or layer 6-specific deletion of *Tbr1*.

## Materials and Methods

### Animals

Floxed TBR1-K228E mice in a C57BL/6J background carrying a knock-in mutation of *Tbr1* (K228E) in exon 1 of the *Tbr1* gene flanked by loxP sites and a neomycin cassette (*Tbr1*^K228E^cassette/+) were designed and generated by Cyagen. The neomycin cassette was removed by crossing *Tbr1*^K228E^cassette/+ mice with protamine-Cre mice (C57BL/6J). WT, *Tbr1*^+/K228E^, and *Tbr1*^K228E /K228E^ mice were genotyped by PCR using the following primers, 5′-TTTTGGAAAAGGGGAATGTG-3′ (forward), 5′-GGAGAAGGGAAGACGTAGGG-3′ (reverse). Animals were housed under a 12-h (13:00–01:00) dark/light cycle and were fed *ad libitum*. The animal study was reviewed and approved by the Committee of Animal Research at Korea Advanced Institute for Science and Technology (KAIST; KA2016-30).

### Structural Modeling of TBR1 and Solubility Prediction

The homology model of TBR1 was built based on the crystal structure of TBX5 (PDB ID: 2X6U) using the SWISS model server (Waterhouse et al., 2018). The DNA-bound form of TBR1 was then constructed by superimposing a homology model of TBR1 onto the crystal structure of TBX3 in complex with DNA (PDB ID 1H6F). The missense mutations, p.K228E and p.N374H, of TBR1 were introduced using a point mutation function in PyMOL software (The PyMOL Molecular Graphics System, version 1.2 r3pre, Schrödinger, LLC), after which energy minimization was performed using Modeler software (Fiser et al., 2000). The stability of TBR1 variants K228E and N374H was predicted by calculating ΔΔG (ΔGWT−ΔGmut) using the DynaMut web server (Rodrigues et al., 2018). All represented structural figures were generated using PyMOL software.

### Protein Expression and Purification

The DNA binding T-box domain (DBD) of the human TBR1 protein with WT sequence (hTBR1_DBD_; 200–400 aa) was subcloned into pET28a vector (Novagen) with enzyme sites, NdeI and HindIII. The K228E point mutation was introduced using the following PCR primers: 5′-ATGATCATCACTGAACAGGGAAGGCGCATGTTT-3′ and 5′- AAACATGCGCCTTCCCTGTTCAGTGATGATCAT-3′. The N374H point mutation was introduced using the following PCR primers: 5′-ACCGCCTACCAGCACACGGATATTACACAACTA-3′ and 5′-TAGTTGTGTAATATCCGTGTGCTGGTAGGCGGT-3′. The hTBR1_DBD_ constructs (WT, K228E, and N374H) transformed in *E. coli* BL21(DE3; Enzynomics) were cultured in Luria-Bertani (LB) media with 30 μg/ml kanamycin at 37°C until OD_600_ reached 0.8, and then the expression of the hTBR1_DBD_ protein was induced by the addition of 0.5 mM isopropyl-β-D-thiogalactoside (IPTG) at 18°C for 16 h. The cultured and harvested cells were ruptured in lysis buffer [20 mM Tirs-HCl pH 7.5, 500 mM NaCl, 5% glycerol, 2 mM β-mercaptoethanol, 30 mM imidazole, and 1 mM phenylmethanesulfonyl fluoride (PMSF)] by sonication and the soluble fractions were collected by centrifuging cell lysate at 20,000 rpm for an hour. The supernatant was loaded to a Ni-NTA column (GE healthcare), which was equilibrated with binding buffer (50 mM Tris-HCl pH 7.5, 500 mM NaCl, 5% glycerol, 30 mM imidazole, and 2 mM β-mercaptoethanol) and hTBR1_DBD_ proteins (WT, K228E, and N374H) were eluted with binding buffer solution containing 250 mM imidazole. The amino terminal hexa-histidine tag was removed by thrombin treatment. The hTBR1_DBD_ proteins lacking the hexa-histidine tag were then further purified using S column (GE healthcare). Fractions containing hTBR1_DBD_ proteins were collected and the buffer was changed against the final buffer (20 mM HEPES pH 7.5, 150 mM NaCl, and 5 mM MgCl_2_) using PD-10 desalting column (GE healthcare).

### Circular Dichroism (CD) for Secondary Structural Comparison

The secondary structures of purified hTBR1_DBD_ proteins (WT, K228E, and N374H) were monitored by circular dichroism (CD) at 25°C in 1 mm cell. The hTBR1_DBD_ protein at the concentration of 150 μM in final buffer (20 mM HEPES pH 7.5, 150 mM NaCl, and 5 mM MgCl_2_) was monitored. CD spectra were recorded using a Jasco J-815 CD spectrometer between 190 and 230 nm at 1-nm intervals averaged over 1 s. The molar ellipticity ([θ]molar) is calculated by equation described below.

$$[\theta ]\text{molar}=\frac{100\times \theta_{\text{obs}}}{d\times m}\deg\times {\text{cm}}^{2}\times {\text{dmol}}^{-1}$$

where, θ_obs_ is the observed value of CD, *d* is distance of measuring cell and *m* is molar concentration of the protein.

### Biolayer Interferometry (BLI)

The oligodexoynucleotides (ODNs) of 5′- biotin- TATATAGGTTGAGTGGCACGTTCCTGGGTGTGAGACC-3′ and 5′- GGTCTCACACCCAGGAACGTGCCACTCAA -3′ were purchased (Macrogen) and annealed in the final buffer. Prior to experiments, all streptavidin biosensors (Fortebio, USA) were hydrated in distilled water at 25°C for 10 min. To avoid buffer effects, the hydrated biosensors were then incubated in kinetic buffer (20 mM HEPES pH 7.5, 100 mM NaCl) at 25°C for 5 min, which showed stable signals. Annealed biotinylated ODNs (10 μM) were immobilized on streptavidin biosensor in kinetic buffer solution for 120 s. The initial baselines were then recorded in a fresh kinetic buffer for 60 s. The association and dissociation sensorgrams of hTBR1_DBD_ WT at concentrations ranging from 2.5 μM to 10 μM were monitored for 120 s each as a positive control by using biolayer interferometry (BLI), BLITZ system (ForteBio, USA). Then, the binding kinetics of hTBR1_DBD_ mutants (K228E and N374H). The equilibrium binding constant (K_d_), association rate constant (k_on_), and dissociation rate constant (k_off_) were determined from the BLI data at various concentrations of the hTBR1_DBD_ protein using the global fitting method provided in data analysis software version 7.0 (Fortebio).

### RNA-Seq Library Preparation and Sequencing

Mouse brains were immersed in RNAlater solution (Ambion, AM7020) to stabilize RNA. RNA extraction, library preparation, and sequencing were performed by Macrogen Incorporation (Seoul, South Korea). RNA samples for sequencing were prepared using a TruSeq RNA Sample Prep Kit v2 (Illumina) according to the manufacturer’s instructions. An Illumina’s HiSeq 4000 were used for sequencing to generate 101-bp paired-end reads. Raw data were submitted to the GEO (Gene Expression Omnibus) repository under accession number GSE134526.

### RNA-Seq Analysis

Transcript abundance was estimated with Salmon (v0.11.2; Patro et al., 2017) in Quasi-mapping-based mode onto the *Mus musculus* genome (GRCm38) with GC bias correction (–gcBias). Quantified gene-level abundance data was imported to R (v.3.5.3) with the tximport (Soneson et al., 2015) package and differential gene expression analysis was carried out using R/Bioconductor DEseq2 (v1.22.2; Love et al., 2014). Normalized read counts were computed by dividing the raw read counts by size factors and fitted to a negative binomial distribution. The *P*-values were first corrected by applying an empirical estimation of the null distribution using the R fdrtool (v.1.2.15) package and then adjusted for multiple testing with the Benjamini–Hochberg correction. Genes with an adjusted *P*-value of less than 0.05 were considered as differentially expressed. Volcano plots were generated using the R ggplot2 (v.3.1.1) package. The Gene Ontology (GO) enrichment analyses were performed using DAVID software (version 6.8; Huang da et al., 2009). Mouse gene names were converted to human homologs using the Mouse Genome Informatics (MGI) database^1^. Gene Set Enrichment Analysis (GSEA^2^, Subramanian et al., 2005) was used to determine whether *a priori*-defined gene sets would show statistically significant differences in expression between WT and *Tbr1* mutant mice. Enrichment analysis was performed using GSEAPreranked (gsea-3.0.jar) module on gene set collections downloaded from Molecular Signature Database (MSigDB) v6.1^3^. GSEAPreranked was applied using the list of all genes expressed, ranked by the fold change and multiplied by the inverse of the *P*-value with recommended default settings (1,000 permutations and a classic scoring scheme). The False Discovery Rate (FDR) was estimated to control the false positive finding of a given Normalized Enrichment Score (NES) by comparing the tails of the observed and null distributions derived from 1,000 gene set permutations. The gene sets with an FDR of less than 0.05 were considered as significantly enriched.

### qRT-PCR

RNA samples were reverse-transcribed using M-MLV cDNA Synthesis kit (Enzynomics, EZ006S) with random hexamer as primers. Synthesized cDNAs were diluted by 5-fold with distilled water and subjected to qPCR reaction using HiPi Real-Time PCR 2x Master Mix (Elpis Bio, EBT-1802) and the following primers: LMBRD1, 5′-CGCCCCTTTAACCTTTAGGCTG-3′ (forward), 5′-AAAGGCCAAAATAGCCAGGAGC-3′ (reverse); ILK, 5′-CTCCCAGTGCTAGGTGCTTG-3′ (forward), 5′-GGTCCACAACGAAATTGGTGC-3′ (reverse); LYPD6, 5′-CATCCCGGGTGCCAGTTCTC-3′ (forward), 5′-GCGTGTCACTCATACAGAGGG-3′ (reverse); PDE3B, 5′-ACTTCACAAGGGATTGAGTGGC-3′ (forward), 5′-GACCTCTTACCACTGCTGCG-3′ (reverse); CRX, 5′-CATCCAGGAGAGTCCCCATTTC-3′ (forward), 5′-TGCTTCCTAGGGGCACTTGAG-3′ (reverse); TBR1, 5′-TGCACGTGGTGGAAGTGAAT-3′ (forward), 5′-CAGCCCGTGTAGATCGTGTC-3′ (reverse); WNT7B, 5′-GCGTGGTCCTACCGCAG-3′ (forward), 5′-GACAATGCTCCGAGCTTCACG-3′ (reverse); RELN, 5′-CAAGCTCAGCGGGTGTCTTA-3′ (forward), 5′-TGCTTACTAGGACGACCTCCAC-3′ (reverse); ADAMTS3, 5′-AAACTTGGGAAGACGAGAGGC-3′ (forward), 5′-AAGGTCCGTGACTTGGCTTC-3′ (reverse); LRPAP1, 5′-ACATCAAGAGCGACACCCTG-3′ (forward), 5′-GGGGCTCTTCAAACTCAGTGG-3′ (reverse); NDNF, 5′-GCTTTTTCCGCACCACACAC-3′ (forward), 5′-CACCAGTAGAACAGCTCCATCCTTA-3′ (reverse); GAPDH, 5′-TCACCACCATGGAGAAGGC-3′ (forward), 5′-GCTAAGCAGTTGGTGGTGCA-3′ (reverse). cDNA templates in qPCR mixture were quantified using BioRad CFX96 Real-Time PCR Detection System (BioRad) with Delta-Delta Ct method in which GAPDH was used as a reference.

### Western Blotting

Whole-brain preparations from WT and *Tbr1^+/K228E^* mice at E17 were extracted and homogenized in the ice-cold lysis buffer using ultrasonicator. Antibodies for immunoblot assays were purchased from commercial sources; TBR1 (Abcam; ab31940); α-tubulin (Sigma Aldrich, T9026).

### Brain Slices for Electrophysiology

For electrophysiology experiments, acute coronal brain slices (300 μm thickness) of WT and *Tbr1^+/K228E^* mice were obtained using a Vibratome (Leica VT1200) after anesthetizing animals with isoflurane (Terrell solution, Piramal healthcare). Brains were extracted and sliced in ice-cold dissection buffer containing (in mM) 212 sucrose, 25 NaHCO_3_, 5 KCl, 1.25 NaH_2_PO_4_, 0.5 CaCl_2_, 3.5 MgSO_4_, 10 D-glucose, 1.25 L-ascorbic acid, and 2 Na-pyruvate bubbled with 95% O_2_/5% CO_2_. The slices were transferred to a recovery chamber at 32°C with normal ACSF (artificial cerebrospinal fluid; in mM: 125 NaCl, 2.5 KCl, 1.25 NaH_2_PO_4_, 25 NaHCO_3_, 10 glucose, 2.5 CaCl_2_, and 1.3 MgCl_2_, oxygenated with 95% O_2_/5% CO_2_). After 30-min recovery at 32°C, slices were recovered for additional 30 min at 20–25°C. For the recording, a single slice was transferred to a submerged-type chamber at 27–28°C with circulating ACSF (2 ml/min) saturated with 95% O_2_ and 5% CO_2_. Recording pipettes were pulled from thin-walled borosilicate glass capillaries (No. 30-0065, Harvard Apparatus) with resistance 2.5–4.0 MΩ using a micropipette electrode puller (PC-10, Narishige).

### Whole-Cell Patch-Clamp Recording

Whole-cell patch-clamp recordings of mPFC layer 6 pyramidal neurons were made using a MultiClamp 700B amplifier (Molecular Devices) and Digidata 1550 (Molecular Devices). During whole-cell patch-clamp recordings, series resistance was monitored for each sweep by measuring the peak amplitude of the capacitance currents in response to short hyperpolarizing step pulse (5 mV, 40 ms). For all electrophysiological measurements described below, layer 6 pyramidal neuron in the prelimbic region of the mPFC were used. Histologically, the pia-to-corpus callosum distance in this region was around 1,500 μm. The cortical region approximately 900–1,200 μm away from pia were used for recordings. After cell rupturing, cells with membrane capacitance larger than 100 were selectively used because a small capacitance is a signature for GABAergic cells.

To measure the intrinsic excitability of mPFC layer 6 pyramidal neurons, recording pipettes (2.5–3.5 MΩ) were filled with an internal solution containing (in mM) 137 K-gluconate, 5 KCl, 10 HEPES, 0.2 EGTA, 10 Na-phosphocreatine, 4 Mg-ATP, and 0.5 Na-GTP, with pH 7.2, 280 mOsm. To measure inhibitory postsynaptic responses, picrotoxin (100 μM), NBQX (10 μM) and D-AP5 (50 μM) were added. After rupturing the cell, currents were clamped, and resting membrane potential (RMP) was measured. Cells with RMP larger than −55 mV were discarded. After stabilizing the cells, RMP was adjusted by −70 mV. To measure the input resistance, hyperpolarizing current steps (1-s duration, 0 to −20 pA, −10 pA step increments) were injected into the patched neurons. To measure the action potential (AP) threshold, a series of current steps (2 ms duration at 2.5 Hz, 0–2,500 pA range, +10 pA increments) were injected until an AP was generated. To obtain the sustained firing rate, a series of current (1-s duration, 0 to +500 pA, +50 pA step increments) were injected. After all measurements, the normal conditions of recorded cells were confirmed by returning to the voltage-clamp mode. In case Ra was >20 in this step, the acquired data was not used.

To measure miniature excitatory postsynaptic currents (mEPSCs) in mPFC layer 6 pyramidal neurons, recording pipettes (3.0–4.0 MΩ) were filled with an internal solution containing (in mM) 100 CsMeSO4, 10 TEA-Cl, 8 NaCl, 10 HEPES, 5 QX-314-Cl, 2 Mg-ATP, 0.3 Na-GTP, and 10 EGTA, with pH 7.25, 295 mOsm. Whole-cell recordings of mEPSCs were made in neurons kept at the holding potential of −70 mV. TTX (1 μM) and picrotoxin (100 μM) were added to ACSF to inhibit spontaneous AP-mediated synaptic currents and inhibitory postsynaptic currents (IPSCs), respectively.

For miniature inhibitory postsynaptic currents (mIPSCs) measurements in mPFC layer 6 pyramidal neurons, recording pipettes (3.0–4.0 MΩ) were filled with an internal solution containing (in mM) 120 CsCl, 10 TEA-Cl, 8 NaCl, 10 HEPES, 5 QX-314-Cl, 4 Mg-ATP, 0.3 Na-GTP and 10 EGTA, with pH 7.35, 280 mOsm. TTX (1 μM), NBQX (10 μM) and D-AP5 (50 μM) were added to ACSF to inhibit spontaneous AP-mediated synaptic currents, AMPAR-mediated currents, and NMDAR-mediated currents, respectively.

For sequential recording of spontaneous EPSCs (sEPSCs) and IPSCs (sIPSCs) in layer 6 pyramidal neurons, recording pipettes (3.0–4.0 MΩ) were filled with an internal solution containing (in mM) 120 CsMeSO_4_, 15 CsCl, 10 TEA-Cl, 8 NaCl, 10 HEPES, 0.25 EGTA 5 QX-314-Cl, 4 Mg-ATP, and 0.3 Na-GTP with pH 7.25–7.35, 280–300 mOsm. In the presence of D-AP5 (50 μM), 2-min recording was started after at least 5 min of stabilization after cell rupture, with −70 mV as the holding potential for sEPSC measurements. After measuring sEPSCs, the holding potential was changed to 0 mV, followed by 1–2 min of stabilization and measurements of sIPSCs for 2 min. The sEPSC and sIPSC data were used for analysis only when the membrane capacitance and resistance are similar to the baseline levels. Data were acquired by Clampex 10.2 (Molecular Devices) and analyzed by Clampfit 10 (Molecular Devices). Drugs were purchased from Abcam (TTX), Tocris (NBQX, D-AP5), and Sigma (picrotoxin).

### Immunohistochemistry

For layer thickness measurement, mouse pups at P5 were deeply anesthetized and decapitated, and brains were removed and incubated in 4% PFA solution at 4°C overnight. Fixed brains were sectioned using Vibratome with 50 μm thickness. Sections were incubated in citrate buffer (pH 6.0) 95°C for 20 min to promote heat-induced epitope retrieval. Subsequently, sections were incubated in 0.1% triton X-100 in PBS (phosphate buffered saline) with 5% normal donkey serum at room temperature for 1 h, and incubated with Tbr1 antibodies (Abcam, ab31940, rabbit, 1:500), Ctip2 antibodies (Abcam, ab18465, rat, 1:500), and Satb2 antibodies (Abcam, ab51502, mouse, 1:500) at 4°C overnight. In the next day, sections were incubated with anti-rat Alexa-488 (Jackson ImmunoResearch, 1:500), anti-rabbit Alexa-568 (Thermofisher, 1:500), anti-mouse Alexa-647 (Jackson ImmunoResearch, 1:500), and DAPI (100 ng/ml) at room temperature for 1 h. Sections were mounted in Dako fluorescence mounting medium. For the counting of somatostatin (SST)-, parvalbumin (Pv)-, and vasoactive intestinal peptide (VIP)-positive interneurons, 3-month-old mice were transcardially perfused with cold 4% paraformaldehyde (PFA) solution and post-fixed in 4% PFA solution at 4°C overnight. Fixed brains were sectioned using Vibratome with 50 μm thickness and stained using somatostatin antibodies (Peninsula, T-4547, rabbit, 1:500), parvalbumin antibodies (Sigma-Aldrich, MAB1572, mouse, 1:500), and VIP antibodies (ImmunoStar, 20077, rabbit, 1:500). Mounted sections were imaged using a Zeiss LSM 780 confocal microscope. For measurements of the thickness of cortical layers, a 63×/1.4 oil-immersion objective was used to image seven consecutive optical sections, and a maximum-intensity-projected image was analyzed using ImageJ. For interneuronal counting, a 20×/0.8 air objective was used to image nine consecutive optical sections, and a z-stacked image was analyzed using 3D object counter plugin of ImageJ.

### Behavioral Assays

All mouse behavioral essays were performed by an experimenter blinded to group-identifying information, and behavioral data were analyzed using EthoVision XT 10 (Noldus), unless indicated otherwise. At least 24-h-long rest periods were given between tests. Behavioral tests were performed in order starting from passive tests, such as measuring home-cage activities, to more stressful tests. Animals were handled for 10 min per day for up to 5 days prior to beginning the battery of behavioral assays so as to reduce stress and anxiety during behavior caused by an experimenter. On each day of a behavioral test, all animals were habituated to a dark room under conditions identical to those of the testing room for 30 min before starting the test.

#### Laboras Test

The Laboratory Animal Behavioral Observation Registration and Analysis System (LABORAS, Metris) was used for long-term monitoring of mouse movements in Laboras cages, conditions similar to those of home cages (Quinn et al., 2003). Mice were individually placed in a single cage within the LABORAS system, and their activities were recorded for 72 consecutive hours. Locomotion, rearing, and self-grooming were measured and automatically analyzed as previously described (Jung et al., 2018; Yoo et al., 2018).

#### Open-Field Test

Animals were placed in a white acryl box (40 × 40 × 40 cm) and video-recorded for 60 min. Light intensity was set to 120 lux. The “center” region was defined as a 20 × 20 cm square in the middle of the arena.

#### Light-Dark Test

The apparatus used for the light-dark test consists of two separate chambers (Light, 21 × 29 × 20 cm; Dark, 21 × 13 × 20 cm) as previously described (Jung et al., 2018). The light chamber was illuminated at 180 lux. The time spent in each chamber was measured.

#### Elevated Plus-Maze Test

Animals were placed in the center region of a plus-arm maze with two open (5 × 30 × 0.5 cm) and closed (5 × 30 × 30 cm) arms. The maze was elevated to a height of 75 cm from the floor. Time spent in open or closed arms and total distance moved were measured.

#### Three-Chamber Test

Social approach and social novelty recognition were assessed by performing three-chamber test (Moy et al., 2004; Silverman et al., 2010) as described previously (Jung et al., 2018; Yoo et al., 2018). Briefly, the apparatus (60 × 40 × 20 cm) consists of three compartments; two side chambers have small containers for either a stranger mouse or a novel object. The subject mouse was placed in the apparatus, leaving the small containers in both compartments empty, and then was allowed to habituate for 10 min. Stranger mouse 1 (129/SvJae strain) was then placed in the container in one side chamber, and a novel object was placed in the container in the other side chamber. The subject mouse was allowed to freely move around the apparatus for 10 min. Social preference towards a new stranger over a familiar stranger was assessed by replacing the object with Stranger mouse 2 (129/SvJae strain) and recording exploration of targets by the mouse for an additional 10 min.

### Dyadic Social Interaction

A direct social-interaction protocol employing a gray Plexiglas box (30 × 30 × 30 cm) was performed to measure social interaction between age-, sex-, and genotype-matched mouse pairs. Briefly, on day 1, each mouse was habituated to the testing conditions by allowing it to freely move around the Plexiglas box for 20 min. On day 2, pairs of unfamiliar age-, genotype-, and sex-matched mice were simultaneously placed in the cage. Mouse behaviors were recorded for 10 min, and the time spent in direct social contacts, including nose-to-nose contact, nose-to-tail contact, following, allo-grooming and other body contacts (Silverman et al., 2010), were analyzed by an experimenter blinded to group-identifying information.

### Pup Ultrasonic Vocalization

Mouse pups at postnatal (P) days P5, P7 and P9 were isolated from their home cages and placed in an empty cage with bedding. The cage was subsequently placed in a soundproof chamber, and ultrasonic vocalizations (USVs) were recorded for 5 min using an ultrasound microphone (Avisoft Bioacoustics). For analysis, spectrograms with a Fourier transformation length of 256, temporal resolution overlap of 75%, and lower cut-off frequency of 25 kHz were generated using Avisoft SASLab Pro software.

### Statistical Analysis

Statistical analyses were performed using GraphPad Prism 7. The normality of data distributions was tested using a D’Agostino and Pearson normality test. Normally distributed data were analyzed using Student’s *t*-test, whereas non-normally distributed data were analyzed using the non-parametric Mann–Whitney test. Differences were considered significant at *p*-values < 0.05. Results are presented as means ± SE. Statistical details are described in Supplementary Table S2.

## Results

### The TBR1-K228E Mutation Inhibits TBR1 Binding to DNA

A previous study on the crystal structure of the T-box domain in complex with DNA from the Brachyury transcription factor in *Xenopus* indicated that the K228 residue in the T-box domain is in direct contact with the DNA backbone (Müller and Herrmann, 1997), and homology modeling of three TBR1 mutations (K389E, W271R and W271C) have been performed (den Hoed et al., 2018). However, the impact of the TBR1-K228E mutation on TBR1-DNA interactions and/or the stability of the core structure of the T-box domain have not been tested by homology modeling or binding experiments.

**Figure 1:**
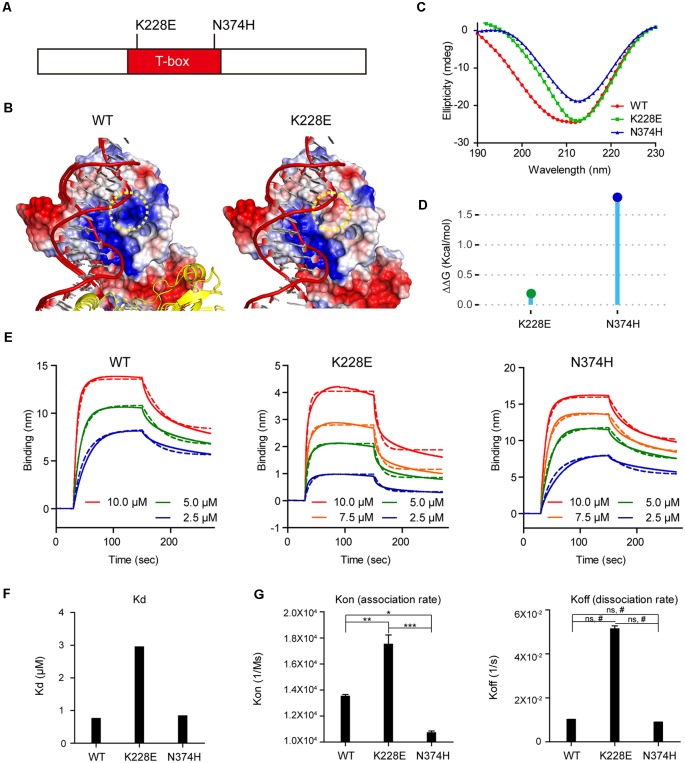
Molecular modeling and measurement of structural stability and DNA-binding affinity of the TBR1-K228E protein.** (A)** Diagram depicting the location of K228E and N374H mutations in the T-box domain of the TBR1 protein. **(B)** Molecular modeling of the TBR1-K228E protein based on the known structure of a complex of the TBR1 T-box domain with DNA. The indicated TBR1-K228E mutation, which converts a positively charged lysine residue (blue) to a negatively charged glutamate residue (red) in a region very close to the phosphodiester backbone of the DNA, likely induces repulsion between the protein and DNA. **(C)** Circular dichroism (CD) profiles of purified TBR1 T-box proteins (WT, K228E, and N374H). Note that the strong negative peaks at ~210 nm indicate that the secondary structures of TBR1 T-box proteins (WT, K228E, and N374H) are largely conserved. **(D)** Prediction of the stability of TBR1 T-box domains (WT, K228E, and N374H) by energy-minimization calculations. Note that both K228E and N374H mutations increase structural stability. **(E–G)** Binding analysis of WT and mutant (K228E and N374H) TBR1 T-box proteins to the *Grin2b*-promoter probe using biolayer interferometry (BLI). The biotinylated *Grin2b*-promoter probe was immobilized on a streptavidin sensor chip, and TBR1 T-box proteins were applied in the mobile phase. The dotted lines, representing fits of the raw data (solid lines), were used to obtain K_d_, K_on_, and K_off_ values. Note the decreased DNA binding by the TBR1-K228E protein, but not the TBR1-N374H protein, compared with WT-TBR1 protein, as indicated by K_d_ and K_on/off_ values. *n* = 3 for WT, K228E, and N274H, *P < 0.05, **P < 0.01, ***P < 0.001, ns, not significant, one-way analysis of variance (ANOVA) with Tukey’s test, ^#^*P* < 0.05, Student’s *t*-test.

We thus first attempted molecular modeling of the T-box domain of human TBR1 using the reported crystal structure of the T-box domain of the DNA-free form of human TBX5 and human TBX3 in complex with DNA to predict potential impacts of the TBR1-K228E mutation. These analyses showed that the TBR1-K228E mutation, which replaces a positively charged lysine with a negatively charged glutamate, induces strong repulsion between the glutamate residue and the adjacent phosphodiester backbone of DNA (Figures 1A,B).

To more quantitatively analyze the reduction in DNA binding of TBR1-K228E, we purified the recombinant T-box domain (aa 200–400) of human wild-type (WT) TBR1 and TBR1-K228E proteins. For comparison, we also generated TBR1-N374H, another TBR1-mutant protein containing an ASD-risk mutation (Neale et al., 2012).

The CD spectra of purified T-box domains of WT-TBR1, TBR1-K228E, and TBR1-N374H indicated that both K228E and N374H mutations altered the conformation and secondary structural composition to a certain degree, although the presence of strong negative peaks at ~210 nm in all purified proteins indicated that secondary structures, including α-helices and β-sheets, were largely conserved (Figure 1C). In addition, normal mode analysis predicted that both K228E and N374H mutations increased the structural stability of the T-box domain compared with that of the WT protein (Figure 1D).

To directly and quantitatively measure the DNA-binding properties of the mutant T-box proteins, we performed BLI, in which a biotinylated *Grin2b*-promoter probe was immobilized on a streptavidin sensor chip, after which purified T-box proteins were applied as mobile analytes and the kinetics of association and dissociation were measured (Figure 1E). These analyses indicated that the K228E mutation caused a strong (~3-fold) decrease in the affinity of the TBR1-K228E protein for DNA, as indicated by an increase in the K_d_ value, whereas the N374H mutation had no effect (Figure 1F). This decrease in the *Grin2b* promoter-binding affinity of the TBR1-K228E protein was associated with a ~2-fold increase in K_on_ but did not involve a significant change in K_off_ (Figure 1G), indicating that the mutation has a greater effect on the association rate than the dissociation rate.

### TBR1-K228E Mice Show Increased TBR1 Protein Levels

**Figure 2:**
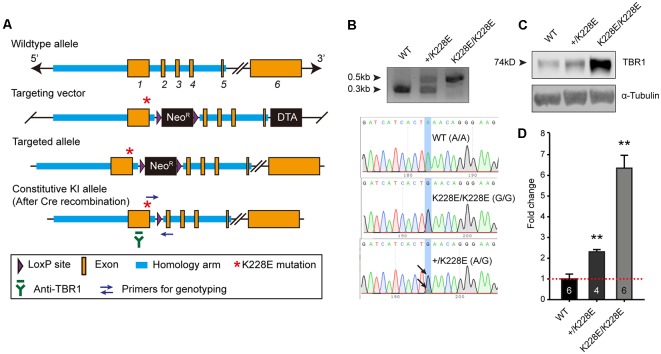
Generation and basic characterization of *Tbr1^+/K228E^* and *Tbr1*^K228E/K228E^ mice. **(A)** *Tbr1* gene knock-in (KI) strategy. Note that the TBR1-K228E mutation is located in exon 1 of the *Tbr1* gene. **(B)** PCR genotyping and confirmation of the TBR1-K228E mutation by DNA sequencing in WT, heterozygous (*Tbr1*^+/K228E^), and homozygous (*Tbr1*^K228E/K228E^) mice (E16.5). **(C,D)** Increased levels of TBR1 protein in *Tbr1*^+/K228E^ and *Tbr1*^K228E/K228E^ brains compared with that in WT brains (E17; males and females), determined by immunoblot analysis of TBR1 protein (~74 kDa) and quantification of TBR1 signals normalized to α-tubulin. The image shown is an example from male mouse samples. Note that levels of the TBR1-K228E protein are strongly increased in a gene dosage-dependent manner. *n* = 6 mice for WT, four mice for Tbr1^+/K228E^, and six mice for Tbr1^K228E/K228E^, **P < 0.01 vs. WT, Mann–Whitney test.

To determine the *in vivo* impacts of the TBR1-K228E mutation, we generated a knock-in (KI) mouse line carrying the TBR1-K228E mutation (Figures 2A,B). Heterozygous and homozygous *Tbr1*-KI mutant mice—*Tbr1*^+/K228E^ and *Tbr1*^K228E/K228E^, respectively—were genotyped by genomic PCR (Figure 2B). *Tbr1*^+/K228E^ mice were born at normal Mendelian ratios, although *Tbr1*^K228E/K228E^ mice could not be detected at the time of genotyping (~P7), similar to the neonatal lethality observed in *Tbr1*-null mice (Hevner et al., 2001).

Intriguingly, whole-brain levels of TBR1 protein were strongly increased in both *Tbr1^+/K228E^* (~2-fold) and *Tbr1*^K228E/K228E^ (~6-fold) mice (Figures 2C,D), in line with the previously reported ~2-fold increase in the stability of the TBR1-K228E protein in heterologous cells (den Hoed et al., 2018). RNA-Seq analyses further showed that this increase in TBR1 protein levels was likely attributable to an increase in the levels of the *Tbr1* transcript (see below).

### The TBR1-K228E Mutation Alters Gene Transcripts Related to Cortical Development

TBR1 is a transcription factor that acts as both a transcriptional activator and repressor (Hsueh et al., 2000; Han et al., 2011). To determine the impacts of the TBR1-K228E mutation on the transcriptomic profile in the developing mouse brain, we performed RNA-Seq analysis using forebrain samples from WT, *Tbr1^+/K228E^*, and *Tbr1*^K228E/K228E^ mice at E16.5, a stage in brain development with strong *Tbr1* expression (Bulfone et al., 1995). Both *Tbr1*^+/K228E^ and *Tbr1*^K228E/K228E^ mice were used in RNA-Seq analysis to test potential gene dosage effects on certain transcripts.

**Figure 3:**
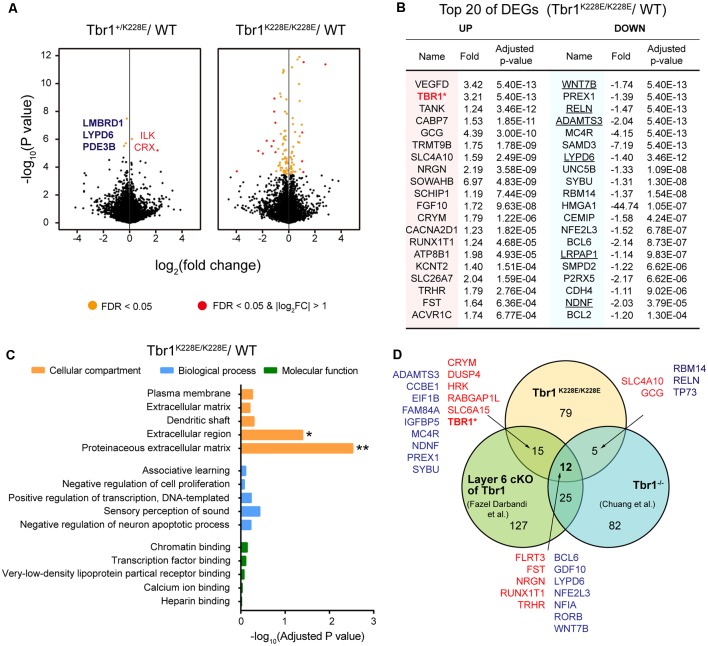
Analysis of differentially expressed genes (DEGs) from *Tbr1^+/K228E^* and *Tbr1*^K228E/K228E^ mice at E16.5.** (A)** Volcano plots showing DEGs from *Tbr1*^+/K228E^ and *Tbr1*^K228E/K228E^ brains (E16.5) relative to WT mouse brains. Names of the DEGs from *Tbr1*^+/K228E^ mice are indicated in the volcano plot, with the three DEGs shared in *Tbr1*^+/K228E^ and *Tbr1*^K228E/K228E^ mice indicated in bold; DEGs from *Tbr1*^K228E/K228E^ mice are indicated in the panel **(B)**. *n* = 5 mice for WT, Tbr1^+/K228E^, and Tbr1^K228E/K228E^, false discovery rate (FDR) < 0.05 (orange); FDR < 0.05 and |log_2_FC| > 1 (red). **(B)** List of the top 40 DEGs (20 up- and 20 downregulated) in the *Tbr1*^K228E/K228E^ brain (E16.5; forebrain). FC, fold change. The underlined gene names indicate DEGs associated with the extracellular matrix (ECM) or extracellular region—the two strongest gene ontology (GO) terms identified in DAVID analyses (see panel **C**). **(C)** David GO analysis of the 111 DEGs from the *Tbr1*^K228E/K228E^ brain (E16.5; adjusted *p*-value < 0.05). Note that significant GO terms were found in the Cellular Component domain, but not in Molecular Function or Biological Process domains. *Adjusted *p*-value < 0.05; **adjusted *p*-value < 0.01. **(D)** Venn diagram showing the overlap between the 111 DEGs from the *Tbr1*^K228E/K228E^ brain at E16.5 and the previously reported 124 DEGs from microarray analyses of the *Tbr1*^−/−^ forebrain at E16.5 (Chuang et al., 2015) and 178 DEGs from mice carrying a layer 6-specific homozygous *Tbr1* deletion (Fazel Darbandi et al., 2018). Note that nearly all up- and downregulated DEGs (red and green, respectively) in the *Tbr1*^K228E/K228E^ brain showed up- and downregulation similar to those of the previous results, with the exception of *Tbr1*, which was upregulated (not downregulated) in the present study. There were no significant associations between the three different groups by Fisher analysis (see Supplementary Table S2 for details).

RNA-seq analyses showed that only five genes—CRX and ILK (upregulated), and LMBRD1, LYPD6, and PDE3B (downregulated)—were differentially expressed between forebrain samples from *Tbr1^+/K228E^* and WT mice at E16.5 (adjusted *p*-value < 0.05), as shown by volcano plots (Figure 3A; Supplementary Table S1). In contrast, homozygous *Tbr1*^K228E/K228E^ brains displayed a relatively large number (111) of differentially expressed genes (DEGs) relative to WT mice, of which 44 were upregulated and 67 were downregulated (Figure 3A). qRT-PCR experiments further indicated the upregulation of Tbr1 and downregulation of Reln in *Tbr1*^K228E/K228E^ brains (Supplementary Figure S1). However, other genes, including the five genes from *Tbr1*^+/K228E^ mice, could not be validated, although the directions of the changes were similar to those observed in RNA-Seq analyses, which might reflect the small changes in the expression of these genes or the higher sensitivity of the RNA-Seq analyses over qRT-PCR analyses.

The top 20 downregulated DEGs among the 111 total DEGs were identified as frequent downstream targets of *Tbr1* or genes that are associated with brain development (Figure 3B). One such gene was *Wnt7b* (Figure 3B), a direct target of *Tbr1* (Huang et al., 2014; Chuang et al., 2015; Fazel Darbandi et al., 2018) that belongs to the Wnt family of secreted signaling proteins known to regulate the development and function of neurons and synapses (Budnik and Salinas, 2011; Mulligan and Cheyette, 2012; Stamatakou and Salinas, 2014). Also identified were *Reln*, a high-risk ASD gene and a direct target of *Tbr1* that encodes a secreted large glycoprotein that regulates cortical development and neuronal migration (Hsueh et al., 2000; Hevner et al., 2001); *Lypd6*, which promotes Wnt signaling through Lrp6 phosphorylation (Ozhan et al., 2013) and calcium conductance in SST-positive interneurons during cortical development (Darvas et al., 2009); and *Bcl6*, a direct target of *Tbr1* (Bedogni et al., 2010; Fazel Darbandi et al., 2018) and marker for the embryonic frontal cortex that functions as a transcriptional repressor to promote neurogenesis by repressing Notch targets (Tiberi et al., 2012; Chiang and Ihrie, 2014). These results suggest that the TBR1-K228E mutation downregulates *Tbr1* target genes that are known to be involved in cortical development.

In addition to downregulated DEGs, the 20 upregulated DEGs were also associated with specific functions. One of the most strongly upregulated genes was 521 *Tbr1* (Figure 3B), in line with the increased levels of TBR1 protein in *Tbr1+/K228E* and 522 *Tbr1K228E/K228E* mice (Figures 2C,D). Other upregulated DEGs included the genes *Nrgn* and *Cacna2d1*, encoding proteins associated with neuronal synapses. *Nrgn*, a putative downstream target of *Tbr1* (Huang et al., 2014; Chuang et al., 2015; Fazel Darbandi et al., 2018), encodes neurogranin, a postsynaptic protein kinase substrate that sets the response threshold to calcium influxes (Gerendasy and Sutcliffe, 1997). *Cacna2d1* encodes alpha-2/delta-1, a voltage-dependent calcium channel subunit that mediates the analgesic action of pregabalin and gabapentin (Field et al., 2006; Xiao et al., 2007) and promotes astrocyte-induced synapse formation (Eroglu et al., 2009; Chung et al., 2015; Geisler et al., 2015).

A DAVID GO analysis using the 111 DEGs from *Tbr1^K228E/K228E^* mice revealed two significant terms, proteinaceous extracellular matrix (ECM) and extracellular region (adjust *p*-value < 0.05), as Cellular Components properties of David GO terms (Figure 3C). The GO term “ECM” was associated with the DEGs, ADAMTS18, COL9A1, WNT7B, NAV2, TNR, CCBE1, RELN, VCAN, ADAMTS3 and NDNF, whereas the GO term “extracellular region” was associated with the DEGs, ADAMTS18, LYPD6, SORL1, NDNF, LRPAP1, COL9A1, HSP90B1, WNT7B, BRINP2, SFRP2, TNR, CCBE1, CEMIP, GDF10, VCAN, RELN, and IGFBP5. Among these, the six underlined genes (WNT7B, ADAMTS3, RELN, LYPD6, NDNF, and LRPAP1) were among the top 20 downregulated DEGs (indicated by underlining in Figure 3B). The ECM has been strongly implicated in the regulation of neural development and synapse formation, function, and plasticity (Venstrom and Reichardt, 1993; Dityatev et al., 2010; Faissner et al., 2010; Wlodarczyk et al., 2011; Frischknecht and Gundelfinger, 2012; Frischknecht et al., 2014; Song and Dityatev, 2018). Therefore, these findings suggest that a homozygous TBR1-K228E mutation decreases the expression of ECM-related genes in mice at E16.5, thereby suppressing neural and synapse development.

Notably, the 111 DEGs from E16.5 *Tbr1^K228E/K228E^* mice overlapped with substantial fractions of the DEGs identified by microarray analysis of *Tbr1*^−/−^ mice (Chuang et al., 2015) and RNA-Seq analysis of *Tbr1*-mutant mice carrying a cortical layer 6-specific homozygous *Tbr1* deletion driven by the *Ntsr1-cre* driver, which is first expressed at ~E16.5 (Fazel Darbandi et al., 2018). Specifically, 17 of the 111 DEGs overlapped with the previously reported 124 DEGs from microarray analyses of *Tbr1*^−/−^ mice at E16.5 (Chuang et al., 2015). In addition, 27 of the 111 DEGs overlapped with the previously reported 178 DEGs from layer 6-specific *Tbr1*-null mice at P5 (Fazel Darbandi et al., 2018; Figure 3D). Intriguingly, a total of 12 genes were found overlapped in all three sets of DEGs, which included upregulated FLRT3, FST, NRGN, RUNX1T1, and TRHR, and downregulated BCL6, GDF10, LYPD6, NFE2L3, NFIA, RORB, and WNT7B.

The DEG analyses on the three *Tbr1*-mutant mouse lines (*Tbr1*^K228E/K228E^, *Tbr1*^−/−^ and layer 6 conditional) differ in multiple aspects, including gene-targeting strategy (knock-in vs. knockout vs. conditional knockout, respectively), brain regions used to prepare mRNA samples (forebrain vs. forebrain vs. cortical layer 6), and developmental stages (E16.5 vs. E16.5 vs. P5). Therefore, these 17 and 27 genes likely play important roles in driving *Tbr1*-mutant phenotypes, both in embryonic (E16.5) and P5 stages. Notably, six of these 27 genes (TBR1, WNT7B, BCL6, MC4R, NFE2L3, and NRGN) were identified by chromatin immunoprecipitation, among other approaches, as direct or putative targets of *Tbr1* in previous studies (Chuang et al., 2015; Notwell et al., 2016; Fazel Darbandi et al., 2018).

### Enrichment of *Tbr1*^+/K228E^ and *Tbr1*^K228E/K228E^ Transcriptomes in Neuron- and Astrocyte-Related Gene Sets

In addition to the DEG analyses of RNA-Seq results from *Tbr1^+/K228E^* and *Tbr1*^K228E/K228E^ mice (E16.5), we attempted a GSEA^4^ (Subramanian et al., 2005), which does not rely on a small number of genes that satisfy certain arbitrary cut-off parameters, such as *p*-values or fold changes. Instead, GSEA uses the entire list of genes ranked according to a certain parameter (i.e., fold change or *p*-value) to evaluate enrichment for precurated gene sets so as to associate a transcriptome with specific biological functions or pathways in an unbiased manner.

**Figure 4:**
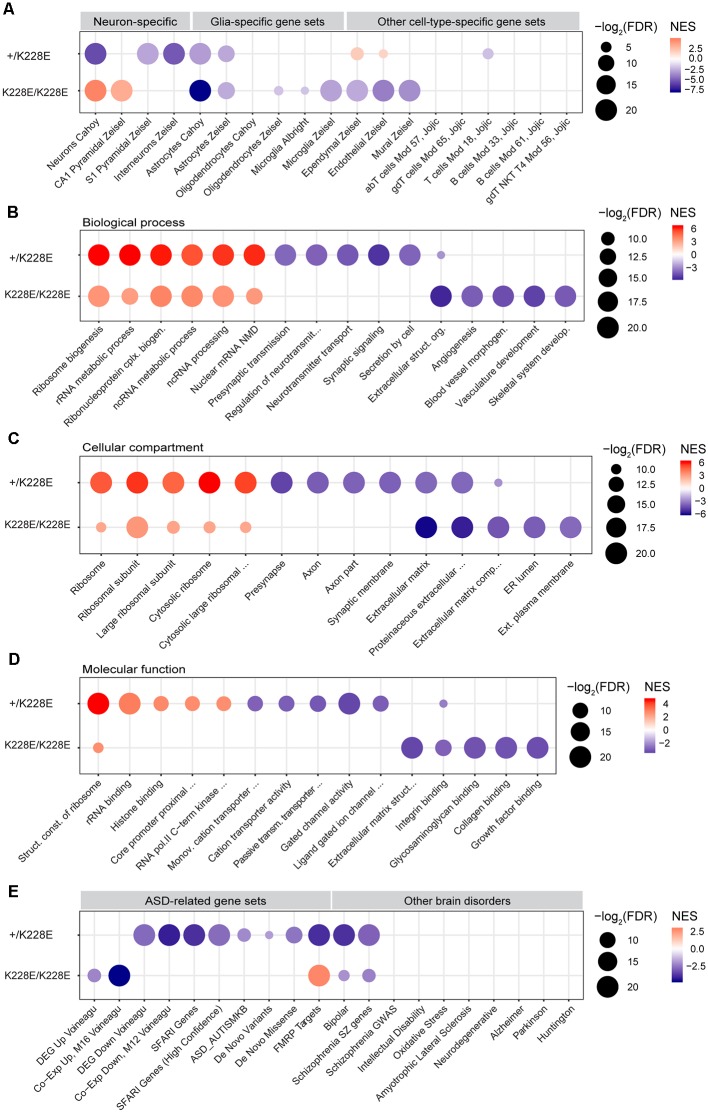
Gene Set Enrichment Analysis (GSEA) of transcriptomes from *Tbr1^+/K228E^* and *Tbr1*^K228E/K228E^ mice at E16.5.** (A)** Negative and dosage-dependent enrichment of *Tbr1*^+/K228E^ and *Tbr1*^K228E/K228E^ transcriptomes in the brain at E16.5 for astrocyte-related gene sets, but distinct enrichments of these lists for gene sets associated with neurons (pyramidal and interneuronal) and other cell types (ependymal, endothelial, and mural). Dot plots were drawn using normalized enrichment score (NES; color of circle) and false detection rate (FDR; intensity of circle) from GSEA results using a one-sided, weighted Smirnov–Kolmogorov test. FDR is the estimated probability that a gene set with a given NES represents a false-positive finding. *n* = 5 mice for WT, HT (heterozygous), and HM (homozygous), FDR < 0.05 (see Supplementary Table S1 for details). **(B–D)** Positive and inverse dosage-dependence of *Tbr1*^+/K228E^ and *Tbr1*^K228E/K228E^ transcriptomes (E16.5) for ribosome-related gene sets, but negative and distinct enrichment of the *Tbr1*^+/K228E^ transcriptome for synapse-related gene sets and the *Tbr1*^K228E/K228E^ transcriptome for ECM-related gene sets. GSEA was performed using precurated GO gene sets in Biological Function **(B)**, Cellular Component **(C)**, and Molecular Function **(D)** domains in the C5 category. The top 4–6 gene sets in each GO domain are indicated. *n* = 5 mice for WT, HT (heterozygous), and HM (homozygous), FDR < 0.05 (see Supplementary Table S1 for details). **(E)** Negative enrichment of the *Tbr1*^+/K228E^ transcriptome for ASD-related gene sets, but no or opposite enrichment of the *Tbr1*^K228E/K228E^ transcriptome for ASD-related gene sets. Note that the *Tbr1*^+/K228E^ transcriptome, but not the *Tbr1*^K228E/K228E^ transcriptome, also shows enrichment for bipolar disease- and schizophrenia-related gene sets. *n* = 5 mice for WT, HT (heterozygous), and HM (homozygous), FDR < 0.05 (see Supplementary Table S1 for details).

To gain insights into the impacts of the TBR1-K228E mutation on gene expression in specific cell types in the brain, we performed GSEA using cell type-specific gene sets, including neurons, pyramidal neurons, interneurons, glial cells (astrocytes, microglia, and oligodendrocytes), and other cell types (i.e., endothelial and ependymal cells), as previously described (Jung et al., 2018). The ranked gene list from heterozygous *Tbr1^+/K228E^* mice at E16.5, hereafter termed the *Tbr1*^+/K228E^ transcriptome, was negatively enriched for gene sets associated with pyramidal neurons, interneurons, and astrocytes (Figure 4A), suggesting underdevelopment of these cell types in *Tbr1*^+/K228E^ embryos at E16.5, consistent with globally suppressed neuroglial development.

Notably, GSEA patterns in the homozygous *Tbr1^K228E/K228E^* brain (E16.5) differed from those in the *Tbr1*^+/K228E^ brain. Specifically, the *Tbr1*^K228E/K228E^ transcriptome was negatively enriched for gene sets associated with astrocytes to a greater extent than that for the *Tbr1*^+/K228E^ transcriptome. In contrast, the *Tbr1*^K228E/K228E^ transcriptome was positively (not negatively) enriched for gene sets associated with neurons generally and CA1 pyramidal neurons specifically (Figure 4A). In addition, the *Tbr1*^K228E/K228E^ transcriptome was negatively enriched for gene sets associated with non-neural cells, such as microglia, ependymal cells, endothelial cells, and mural cells.

Thus, the *Tbr1^K228E/K228E^* transcriptome is similar to the *Tbr1*^+/K228E^ transcriptome with respect to the negative enrichment for astrocyte gene sets but differs from the *Tbr1*^+/K228E^ transcriptome in that it is positively enriched for neuron-related gene sets and negatively enriched for other cell type-related gene sets. These differences between heterozygous and homozygous TBR1-K228E mutations reveal both gene dosage-dependent and -independent effects on gene expression.

### Enrichment of *Tbr1*^+/K228E^ and *Tbr1*^K228E/K228E^ Transcriptomes in Ribosome-, Synapse- and ECM-Related Gene Sets

To gain insight into the impacts of heterozygous and homozygous *Tbr1* deletion on the transcription of genes associated with biological pathways and cellular and molecular functions, we performed GSEA using gene sets in the C5 GO category. Intriguingly, the *Tbr1*^+/K228E^ transcriptome (E16.5) was strongly and positively enriched for multiple ribosome-related gene sets in Biological Process, Cellular Component, and Molecular Function domains (Figures 4B–D). The *Tbr1*^K228E/K228E^ transcriptome was also positively enriched for ribosome-related gene sets, although to a lesser extent than that of the *Tbr1*^+/K228E^ transcriptome. These results suggest the possibility that a *Tbr1* deletion might drive stronger protein translation, although this tendency is weaker in the case of the homozygous mutation, indicative of an inverse gene dosage effect. The stronger enrichment of the *Tbr1*^+/K228E^ transcriptome for ribosome-related gene sets relative to the *Tbr1*^K228E/K228E^ transcriptomes contrasts with the fewer number of DEGs in the *Tbr1*^+/K228E^ transcriptome, suggesting the usefulness of GSEA using the whole transcriptome in cases where the numbers of DEGs are small.

In contrast to its positive enrichment for ribosome-related gene sets, the *Tbr1^+/K228E^* transcriptome was negatively enriched for synapse-related gene sets, including presynaptic structures and functions and ligand-gated ion channels (Figure 4C). The *Tbr1*^K228E/K228E^ transcriptome, however, was not negatively enriched for synapse-related gene sets but did show negative enrichment for gene sets associated with the ECM (Figures 4B–D), in line with the strong enrichment of ECM-related GO terms among the 111 DEGs from *Tbr1*^K228E/K228E^ mice (Figure 3C). Collectively, these findings suggest that heterozygous and homozygous *Tbr1* deletions strongly and similarly promote expression of ribosome-related genes, but distinctly suppress expression of synapse- and ECM-related genes, respectively.

### Enrichment of ASD-Related Gene Sets Among *Tbr1*^+/K228E^ and *Tbr1*^K228E/K228E^ Transcriptomes

*TBR1* is a high-risk ASD gene and has been shown to affect the expression of many other ASD-risk genes (Neale et al., 2012; O’Roak et al., 2012, 2014; Traylor et al., 2012; De Rubeis et al., 2014; Deriziotis et al., 2014; Hamdan et al., 2014; Palumbo et al., 2014; Chuang et al., 2015; Sanders et al., 2015; Notwell et al., 2016; Bowling et al., 2017; Geisheker et al., 2017; Fazel Darbandi et al., 2018; McDermott et al., 2018; Vegas et al., 2018). We thus tested whether the *Tbr1*^+/K228E^ and *Tbr1*^K228E/K228E^ transcriptomes are enriched for ASD-related gene sets (Werling et al., 2016). We also assessed gene sets associated with other neurological and psychiatric brain disorders.

The *Tbr1^+/K228E^* transcriptome (E16.5) was negatively and strongly enriched for multiple ASD-related gene sets, including DEG Down Voineagu and Co-Exp Down, M12 Voineagu, SFARI genes, ASD_AUTISMKB, De Novo Variants, De Novo Missense, and FMRP Targets, whereas no enrichment was observed for gene sets upregulated in ASD (DEG Up Voineagu and Co-Exp Up, M16 Voineagu; Figure 4E). In addition, the *Tbr1*^+/K228E^ transcriptome was enriched for gene sets associated with bipolar disorder and schizophrenia, but not for gene sets associated with other brain disorders. These results suggest that the *Tbr1*^+/K228E^ transcriptome (E16.5) strongly mimics the transcriptomic pattern of ASD.

The transcriptome from homozygous *Tbr1^K228E/K228E^* mice (E16.5), however, showed little enrichment for ASD-related gene sets (Figure 4E). Instead, it was negatively enriched for gene sets upregulated in ASD (DEG Up Voineagu and Co-Exp Up, M16 Voineagu), a pattern opposite that observed in ASD. Moreover, the *Tbr1*^K228E/K228E^ transcriptome was positively enriched for the FMRP targets gene set; again, an enrichment pattern opposite that observed in ASD.

These results collectively suggest that the *Tbr1^+/K228E^* transcriptome strongly mimics the transcriptomic changes observed in ASD, whereas the *Tbr1*^K228E/K228E^ transcriptome does not, suggesting the possibility of compensatory changes in the context of the stronger homozygous TBR1-K228E mutation.

### Altered Numbers of Parvalbumin-Positive Neurons in Superficial and Deep Cortical Layers of the *Tbr1*^+/K228E^ mPFC

TBR1 is a critical regulator of cortical development (Bulfone et al., 1995, 1998; Dwyer and O’Leary, 2001; Hevner et al., 2001, 2002; Englund et al., 2005; Kolk et al., 2006; Bayatti et al., 2008; Han et al., 2011; McKenna et al., 2011; Cánovas et al., 2015; Marinaro et al., 2017; Elsen et al., 2018; Liu et al., 2018). We thus assessed possible impairments in various aspects of cortical development, including cortical thickness, layer organization and cellular distribution in the *Tbr1^+/K228E^* brain.

**Figure 5:**
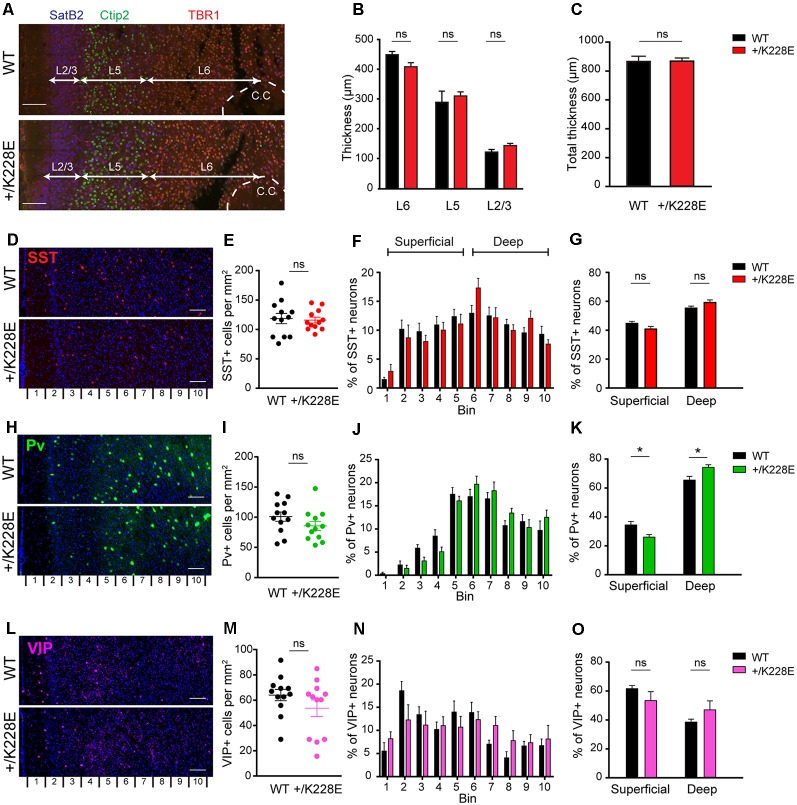
Altered numbers of parvalbumin (Pv)-positive interneurons in superficial and deep cortical layers, but normal cortical thickness in the *Tbr1^+/K228E^* mPFC. **(A–C)** Normal thickness of the whole cortex and individual cortical layers in the prelimbic region of the mPFC in *Tbr1*^+/K228E^ (HT) mice (P5), as indicated by the thickness of total **(C)** and individual **(B)** cortical layers. Layers were marked by immunofluorescence staining: layer2/3, SatB2; layer 5/6, Ctip2; and layer 6, TBR1. *n* = 3 sections from three mice, ns, not significant, Mann–Whitney test. Scale bar, 100 μm. **(D–G)** Normal total number of somatostatin (SST)-positive neurons in the prelimbic region of the mPFC of *Tbr1*^+/K228E^ mice (HT; 3 months), as indicated by the number of SST-positive cells across the total cortical depth (bin 1–10; lower numbers correspond to upper layers) and the sum of superficial and deep cortical layers (bins 1–5 and 6–10 for superficial and deep layers, respectively). *n* = 12 sections from three mice for WT and HT, ns, not significant, two-way ANOVA with Sidak’s multiple comparison test (for SST-positive cells in superficial or deep layer) and Student’s *t*-test (total SST-positive cells). Scale bar, 100 μm. **(H–K)** Decreased numbers of Pv-positive neurons in superficial layers and increased numbers in deep cortical layers in the prelimbic region of the mPFC of *Tbr1*^+/K228E^ (HT) mice (3 months). *n* = 12 sections from three mice for WT and HT, *P < 0.05, ns, not significant, two-way ANOVA with Sidak’s multiple comparison test (for PV-positive cells in superficial or deep layers) and Student’s *t*-test (total PV-positive cells). Scale bar, 100 μm. **(L–O)** Normal total number of vasoactive intestinal peptide (VIP)-positive neurons in the prelimbic region of the mPFC of *Tbr1*^+/K228E^ (HT) mice (3 months), as indicated by the number of VIP-positive cells across the total cortical depth. *n* = 12 sections from three mice for WT and HT, ns, not significant, two-way ANOVA with Sidak’s multiple comparison test (for VIP-positive cells in superficial or deep layer) and Student’s *t*-test (total VIP-positive cells). Scale bar, 100 μm.

We first measured the thickness and layer distribution of the neocortex in the prelimbic region of the medial prefrontal cortex (mPFC) in *Tbr1^+/K228E^* mice at P5, a developmental stage at which neurons in different cortical layers can be readily visualized by immunostaining. There was no statistically significant difference in the thickness of the cortex as a whole or of individual cortical layers (L2/3, L5, and L6) between WT and *Tbr1*^+/K228E^ mice, visualized by immunofluorescence staining for specific layers [SatB2, mainly layer 2/3; Ctip2, layer 5/6 (weakly for 6); TBR1, layer 6; Figures 5A–C; Supplementary Figure S2].

We next counted SST-, Pv-, and VIP-positive GABAergic interneurons across the depth of the cortex at 3 months, the developmental stage at which we performed electrophysiological and behavioral experiments (see below). These counts revealed no differences in the total numbers of SST-, Pv-, or VIP-positive interneurons between WT and *Tbr1^+/K228E^* mice (Figures 5E,I,M). Intriguingly, however, the number of Pv-positive interneurons in superficial cortical layers was decreased, whereas that in the deep layers was increased (Figures 5J,K). Although SST- and VIP-positive neurons showed a similar trend toward reciprocal changes in superficial and deep layers, these differences did not reach statistical significance (Figures 5F,G,N,O).

These results collectively suggest that the heterozygous TBR1-K228E mutation does not affect the thickness of the whole cortex or individual cortical layers, but does affect the number of Pv-positive interneurons in both superficial (decreased) and deep (increased) cortical layers.

### Increased Inhibitory Synaptic Transmission in Cortical Layer 6 Pyramidal Neurons

The increased number of Pv-positive interneurons in cortical layer 6 in the *Tbr1^+/K228E^* brain points to the possibility of altered synaptic transmission in layer 6 pyramidal neurons. Accordingly, we next measured excitatory and inhibitory transmission in these neurons; we also measured neuronal excitability, a property that bidirectionally impacts synaptic transmission.

To this end, we first measured mEPSCs in layer 6 pyramidal neurons in the prelimbic region of the mPFC in WT and *Tbr1^+/K228E^* brains (11–12 weeks). We used young adult mice for these experiments to allow direct comparisons with electrophysiological and behavioral experiments performed in mice of this age (see below).

**Figure 6:**
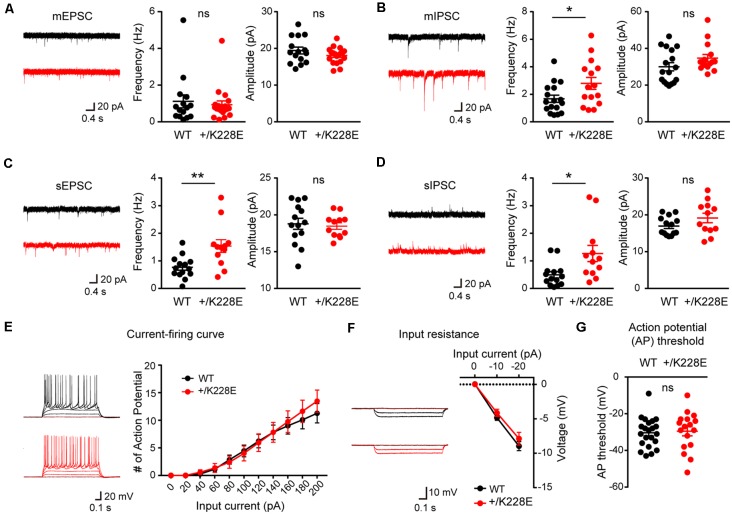
Increased inhibitory synaptic transmission in cortical layer 6 pyramidal neurons.** (A)** Normal miniature excitatory postsynaptic current (mEPSC) frequency and amplitude in layer 6 pyramidal neurons in the prelimbic region of the mPFC of *Tbr1^+/K228E^* (HT) mice (11–12 weeks). *n* = 15 neurons from three mice for WT and 19 neurons from four mice for HT, ns: not significant, Student’s *t*-test (mEPSC amplitude), Mann–Whitney test (mEPSC frequency). **(B)** Increased frequency but normal amplitude of miniature inhibitory postsynaptic currents (mIPSCs) in layer 6 pyramidal neurons in the prelimbic region of the mPFC of *Tbr1*^+/K228E^ (HT) mice (11–12 weeks). *n* = 17 neurons from three mice for WT and 15 neurons from three mice for HT, *P < 0.05, ns: not significant, Mann–Whitney test (mIPSC amplitude), Student’s *t*-test (mIPSC frequency). **(C)** Increased frequency but normal amplitude of spontaneous EPSCs (sEPSCs) in layer 6 pyramidal neurons in the prelimbic region of the mPFC of *Tbr1*^+/K228E^ (HT) mice (11–12 weeks). *n* = 14 neurons from three mice for WT and 12 neurons from three mice for HT, **P < 0.01, ns: not significant, Mann–Whitney test (sEPSC frequency), Student *t*-test (sEPSC amplitude). **(D)** Increased frequency but normal amplitude of spontaneous IPSCs (sIPSCs) in layer 6 pyramidal neurons in the prelimbic region of the mPFC of *Tbr1*^+/K228E^ (HT) mice (11–12 weeks). *n* = 14 neurons from three mice for WT and 12 neurons from three mice for HT, *P < 0.05, ns: not significant, Mann–Whitney test (sIPSC frequency and amplitude). **(E–G)** Normal excitability of layer 6 pyramidal neurons in the prelimbic region of the mPFC in *Tbr1*^+/K228E^ (HT) mice (11–12 weeks), as indicated by current-firing curve **(E)**, input resistance **(F)**, and action potential (AP) threshold **(G)**. *n* = 22 neurons from four mice for WT and 17 neurons from four mice for HT, ns: not significant, current-firing curve: two-way repeated-measures ANOVA with Sidak’s multiple comparisons test, AP threshold: student’s *t*-test, input resistance: two-way repeated measures ANOVA with Sidak’s multiple comparisons test.

There were no differences in the frequency or amplitude of mEPSCs between genotypes (Figure 6A). In contrast, there was a significant increase in the frequency, but not amplitude, of mIPSCs in *Tbr1^+/K228E^* layer 6 pyramidal neurons (Figure 6B). These results suggest that inhibitory, but not excitatory, synaptic transmission onto mPFC layer 6 pyramidal neurons were selectively increased by a heterozygous TBR1-K228E mutation.

Because network activities often affect excitatory and inhibitory synaptic transmission, we next measured sEPSCs and sIPSCs in *Tbr1^+/K228E^* mPFC layer 6 pyramidal neurons in the absence of tetrodotoxin to allow AP firings. Intriguingly, there was an increase in the frequency, but not amplitude, of sEPSCs (Figure 6C), a finding that contrasts with the normal frequency of mEPSCs. The frequency, but not amplitude, of sIPSCs was also increased in these mutant neurons (Figure 6D), similar to the observed increased mIPSC frequency. These results suggest that network activity has no effect on increased inhibitory synaptic transmission, but induces an increase in the frequency of excitatory synaptic transmission, an effect that likely serves to normalize the balance between synaptic excitation and inhibition (E/I balance).

Lastly, we measured neuronal excitability in *Tbr1^+/K228E^* mPFC layer 6 pyramidal neurons. We found no difference in the excitability of *Tbr1*^+/K228E^ neurons between genotypes, as shown by the current-firing curve, input resistance, and AP threshold (Figures 6E–G).

These results collectively suggest that the heterozygous TBR1-K228E mutation leads to an abnormal increase in inhibitory synaptic transmission in layer 6 pyramidal neurons without affecting excitatory synaptic transmission or neuronal excitability. This change (increased inhibitory transmission) in the presence of network activity leads to an increase in excitatory transmission, a compensatory change that likely acts to maintain a normal synaptic E/I balance.

### ASD-Like Social Deficits, Increased Repetitive Behaviors, Altered Anxiety-Like Behavior, and Modestly Increased Locomotion in *Tbr1*^+/K228E^ Mice

Because *Tbr1* is strongly associated with ASD (Bedogni et al., 2010; Neale et al., 2012; O’Roak et al., 2012, 2014; Traylor et al., 2012; De Rubeis et al., 2014; Deriziotis et al., 2014; Hamdan et al., 2014; Palumbo et al., 2014; Chuang et al., 2015; Sanders et al., 2015; Bowling et al., 2017; Geisheker et al., 2017; McDermott et al., 2018; Vegas et al., 2018), we subjected *Tbr1*^+/K228E^ mice first to ASD-related behavioral tests.

**Figure 7:**
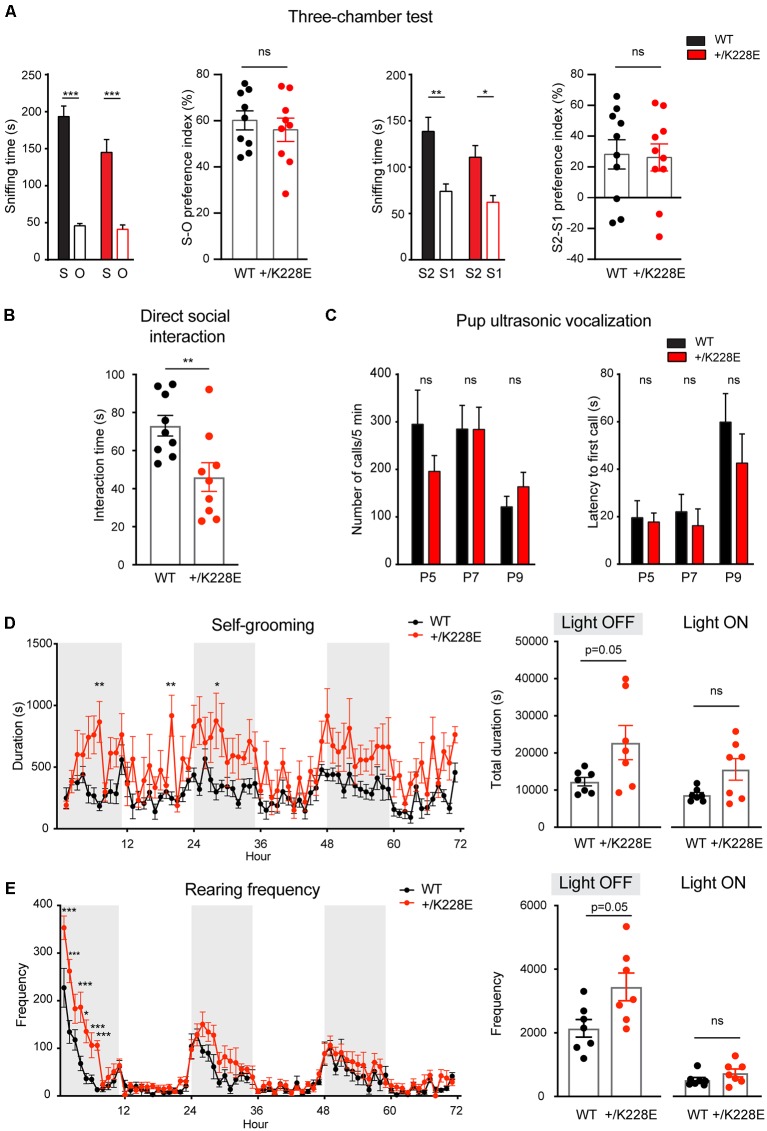
Social deficits and repetitive behaviors in *Tbr1^+/K228E^* mice.** (A)** Normal levels of social approach and social novelty recognition in *Tbr1*^+/K228E^ mice (3 months; male) in the three-chamber test, as shown by time spent exploring social (S1) vs. object (O) targets for social approach, or old stranger (S1) vs. new stranger (S2) for social novelty, and the social preference index [i.e., (time spent in sniffing S1 − time spent in O)/total time spent (S1+O) × 100]. *n* = 9 mouse pairs for WT and HT (social approach), *n* = 10 mouse pairs for WT and HT (social novelty), ***P < 0.001, two-way ANOVA with Sidak’s test (for S-O or S2-S1 comparisons; multiple comparisons between S-S, S1-S1, or S2-S2 were not performed for the lack of genotype × target interactions), ns: not significant, Student’s *t*-test (preference index). **(B)** Suppressed direct social interaction in *Tbr1*^+/K228E^ mice (3 months; male), as indicated by time spent in total interaction between age-, sex- and genotype-matched mouse pairs. *n* = 9 mouse pairs for WT and HT, **P < 0.01, ns: not significant, Mann–Whitney test. **(C)** Normal ultrasonic vocalizations (USVs) in *Tbr1*^+/K228E^ pups (P5–9; male and female) separated from their mothers, as indicated by the number of emitted USVs and latency to first USV call. *n* = 12 pups (WT-P5), 9 (HT-P5), 15 (WT-P7), 16 (HT-P7), 15 (WT-P9) and 16 (HT-P9; number of calls), *n* = 12 (WT-P5), 9 (HT-P5), 15 (WT-P7), 15 (HT-P7), 15 (WT-P9) and 16 (HT-P9; latency to first call), ns, not significant, two-way ANOVA with Sidak’s multiple comparisons test. **(D,E)** Enhanced self-grooming and rearing in *Tbr1*^+/K228E^ mice (3 months; male) in Laboras cages, where mouse movements were monitored for 72 consecutive hours without prior habituation. *n* = 7 mice for WT and HT, *P < 0.05, ***P < 0.001, ns: not significant, two-way repeated-measures ANOVA with Sidak’s multiple comparison test and Mann–Whitney test (light-on/off).

In the three-chamber test for social interaction (Crawley, 2004; Moy et al., 2009; Silverman et al., 2010), *Tbr1^+/K228E^* mice (3 months; male) exhibited normal social approach and social novelty recognition, as shown by the time spent exploring social or object targets and the social preference index (Figure 7A). However, *Tbr1*^+/K228E^ mice exhibited reduced social interaction in direct social-interaction tests, as shown by the total time spent in social interaction (Figure 7B). Upon separation from their mothers, *Tbr1*^+/K228E^ pups (P5–9; male and female) showed normal levels of social communication by USVs, a measure of social communication in rodents (Scattoni et al., 2009; Burgdorf et al., 2011; Wöhr and Schwarting, 2013; Portfors and Perkel, 2014), as indicated by the number of emitted USVs and the latency to the first USV call (Figure 7C). These results are suggestive of normal levels of anxiety-like behaviors and social communication in *Tbr1*^+/K228E^ mice.

In tests for repetitive behaviors using Laboras cages, in which mouse movements were monitored for 72 consecutive hours, *Tbr1^+/K228E^* mice showed modestly increased repetitive self-grooming during the light-off period, but not during the light-on period (Figure 7D). Measurements of rearing, a form of exploratory behavior, in Laboras cages, revealed a significant increase in *Tbr1*^+/K228E^ mice during the light-off period, but not during the light-on period (Figure 7E). These results suggest increased repetitive self-grooming and rearing in *Tbr1*^+/K228E^ mice.

**Figure 8:**
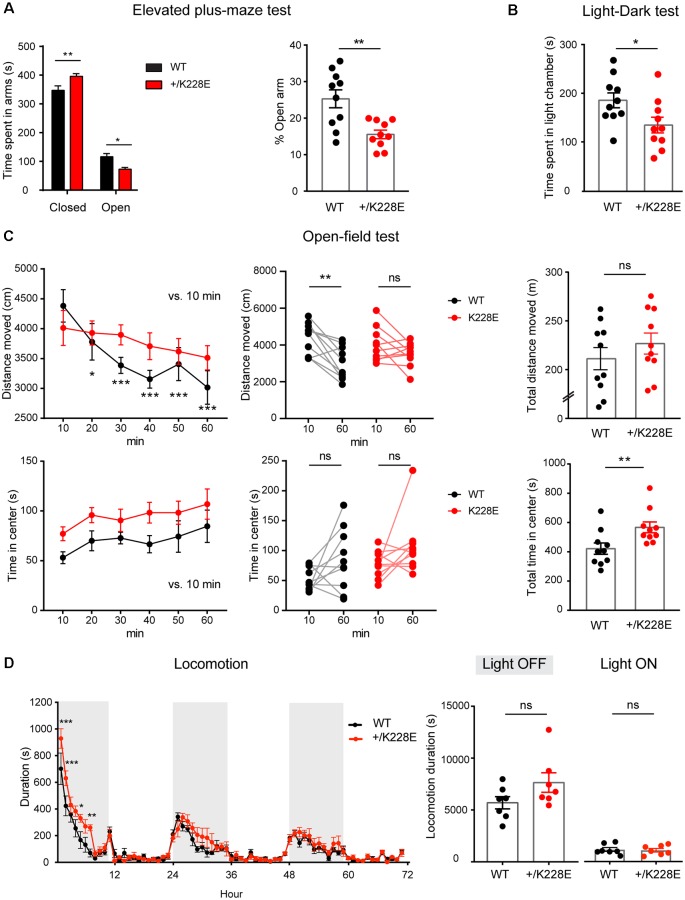
Altered anxiety-like behavior and modestly increased locomotor activity in *Tbr1^+/K228E^* mice. **(A)** Enhanced anxiety-like behavior in *Tbr1*^+/K228E^ mice (3 months; male) in the elevated plus-maze test, as shown by time (total and %) spent in open/closed arms. *n* = 10 mice for WT and HT, *P < 0.05, **P < 0.01, two-way ANOVA with Sidak’s multiple comparison test (total time) and Mann–Whitney test (% time). **(B)** Enhanced anxiety-like behavior in *Tbr1*^+/K228E^ mice (3 months; male) in the light-dark test, as shown by time spent in the light chamber. *n* = 10 mice for WT and HT, *P < 0.05, Mann–Whitney test. **(C)** Normal locomotor activity but decreased habituation in *Tbr1*^+/K228E^ mice (3 months; male) in the open-field test. Note that the time spent in the center region of the open-field arena is abnormally increased, indicative of anxiolytic-like behavior, although the suppressed habitation to the environment, indicated by the increasing difference in the distance moved across the 10-min sections and the distance moved during the first and last 10 mins, might complicate this interpretation. *n* = 10 mice for WT and HT, *P < 0.05, **P < 0.01, ***P < 0.001, ns: not significant, repeated measures two-way ANOVA with Dunnett’s multiple comparisons test (Distance moved over 60 min, Center time over 60 min), Wilcoxon matched-pairs signed-rank test (10 min vs. 60 min), Student’s *t*-test (total distance moved), and Mann–Whitney test (total time in center). **(D)** Modestly increased locomotor activity in *Tbr1*^+/K228E^ mice (3 months; male) in Laboras cages. Note that hyperactivity is mainly observed during the first ~6 h of the light-off period on day 1. *n* = 7 mice for WT and HT, *P < 0.05, **P < 0.01, ***P < 0.001, ns: not significant, two-way repeated-measures ANOVA with Sidak’s multiple comparisons test and Mann–Whitney test (light-on/off).

Finally, given that ASD is associated with anxiety and attention-deficit/hyperactivity disorder (ADHD)-like hyperactivity, we measured anxiety-like and locomotor behaviors in *Tbr1^+/K228E^* mice. In the elevated plus-maze test, *Tbr1*^+/K228E^ mice spent more time in closed arms, but less time in open arms (Figure 8A), indicative of anxiety-like behavior. In addition, *Tbr1*^+/K228E^ mice spent less time in the light chamber in the light-dark test (Figure 8B), further indicative of anxiety-like behavior. These results suggest that *Tbr1*^+/K228E^ mice show increases in anxiety-like behaviors.

In the open-field test, *Tbr1^+/K228E^* mice showed normal levels of locomotor activity (Figure 8C). Notably, *Tbr1*^+/K228E^ mice spent an increased amount of time in the center, indicative of anxiolytic-like behavior. However, *Tbr1*^+/K228E^ mice also showed reduced habituation to the novel open-field environment (Figure 8C), which is known to involve anxiety (Bailey and Crawley, 2009; Campbell et al., 2014) and may contribute to the increased time spent in the center region of the open-field arena. In Laboras cages, however, *Tbr1*^+/K228E^ mice showed modest hyperactivity during the first ~6 h on day 1 (light-off period), although total locomotion over 3 days did not change in light-off or light-on periods (Figure 8D).

Taken together, these results suggest that *Tbr1^+/K228E^* mice display social deficits, repetitive behaviors, altered anxiety-like behavior, and modestly increased locomotion.

## Discussion

In the current study, we evaluated impacts of the ASD-derived TBR1-K228E mutation in mice, identifying a multitude of changes at transcriptomic, protein, cellular, synaptic, and behavioral levels.

Our molecular modeling data indicate that the TBR1-K228E mutation leads to a strong change in local charges, from positive to negative, on the surface of the TBR1 protein near the region that binds the negatively charged backbone of target DNA (Figure 1B). While this has a minimal impact on secondary structures or the structural stability of the protein (Figures 1C,D), it leads to approximately a 3-fold decrease in the K_d_ value for binding of the mutant protein to the *Grin2b*-promoter region, reflecting predominantly a decrease in the dissociation rate (Figures 1E–G). These changes are predicted to lead to decreased TBR1-dependent regulation of the expression of target genes.

Interestingly, whole-brain levels of TBR1 protein were markedly increased in *Tbr1^+/K228E^* mice (~2.5-fold) and *Tbr1*^K228E/K228E^ mice (~6-fold; Figures 2C,D). These increases are reminiscent of the previous reported increase in the stability of the TBR1-K228E protein in HEK293 cells (den Hoed et al., 2018). Given that levels of the *Tbr1* transcript were increased, whereas expression of known *Tbr1* target genes such as *Wnt7b*, *Reln*, and *Bcl6* were decreased in *Tbr1*^K228E/K228E^ mice (Figures 3A,B), the increased levels of TBR1 protein likely reflect a compensatory upregulation of *Tbr1* attributable to the limited binding of the TBR1-K228E protein to target DNAs (Figure 1).

*Tbr1^+/K228E^* and *Tbr1*^K228E/K228E^ mice at E16.5 showed altered transcriptomic profiles relative to WT mice (Figures 3, 4). These changes may reflect the limited interaction of TBR1-K228E protein with FOXP2 (Deriziotis et al., 2014), a transcription factor critical for brain development that may act together with TBR1 to regulate gene transcription. In addition, overexpressed TBR1-K228E protein in excitatory neurons may be normally targeted to the nucleus, but could form abnormal aggregates with TBR1 binding partners known to be important for transcriptional regulation, such as CASK or FOXP2, thereby inhibiting their functions in the nucleus in a dominant-negative manner, as observed in HEK293 cells and suggested previously (Deriziotis et al., 2014).

The genes whose expression was altered in *Tbr1^+/K228E^* and *Tbr1*^K228E/K228E^ mice at E16.5 were associated with diverse functions. In particular, genes that were differentially expressed between *Tbr1*^K228E/K228E^ and WT mice were strongly associated with the ECM (Figure 3C). In addition, GSEA results indicated strong, negative enrichment of ECM-related gene sets in the *Tbr1*^K228E/K228E^ transcriptome, but not the *Tbr1*^+/K228E^ transcriptome (Figure 4B). These results suggest that the homozygous TBR1-K228E mutation leads to strong suppression of the expression of ECM-related genes, which are known to regulate neural development and synapse formation, function, and plasticity (Venstrom and Reichardt, 1993; Dityatev et al., 2010; Faissner et al., 2010; Wlodarczyk et al., 2011; Frischknecht and Gundelfinger, 2012; Frischknecht et al., 2014; Song and Dityatev, 2018). Notably, our GSEA data on the *Tbr1*^K228E/K228E^ transcriptome indicated that gene sets associated with astrocytes, microglia, ependymal cells, endothelial cells, and mural cells were negatively enriched (Figure 4A). Therefore, the limited expression of ECM-related genes in *Tbr1*^K228E/K228E^ mice at E16.5 might be a reflection of suppressed development of multiple cell types, including astrocytes, in the mutant embryo. Notably, the negative enrichment for gene sets associated with astrocytes is at odds with a previous report that TBR1 promotes neuronal differentiation and suppresses astrocyte formation in the olfactory bulb (Méndez-Gómez et al., 2011), although whether TBR1 has a different role in the mPFC remains to be determined.

DEG and GSEA analyses revealed shared and distinct changes in *Tbr1^+/K228E^* and *Tbr1*^K228E/K228E^ transcriptomes at E16.5. However, because such a small number of DEGs were identified between *Tbr1*^+/K228E^ and WT mice (five), it was not possible to make a meaningful quantitative comparison of *Tbr1*^+/K228E^ and *Tbr1*^K228E/K228E^ transcriptomes based on this analysis. In contrast, GSEA results provided a clear answer to this question. Specifically, the *Tbr1*^+/K228E^ and *Tbr1*^K228E/K228E^ transcriptomes showed shared negative enrichment for astrocyte-related gene sets and positive enrichment for ribosome-related gene sets, although the latter displayed an inverse gene-dosage effect, unlike the former. In contrast, the *Tbr1*^+/K228E^ (but not *Tbr1*^K228E/K228E^) transcriptome showed unique, negative enrichment for gene sets associated with neurons, synapses, and ASD risk. On the other hand, the *Tbr1*^K228E/K228E^ transcriptome (but not the *Tbr1*^+/K228E^ transcriptome) showed unique, negative enrichment for gene sets associated with specific non-neuroglial cells (i.e., ependymal and endothelial), the ECM, and ASD. It is unclear why the *Tbr1*^+/K228E^ transcriptome is negatively enriched for neuron/astrocyte-related and synapse-related gene sets, but positively associated with ribosome-related gene sets. It is conceivable that the strong suppression of neuroglia- and synapse-related gene expression might promote strong compensatory expression of ribosome-related genes, which are known to be important for neuronal outgrowth and differentiation of synapses during brain development and maintenance (Bramham and Wells, 2007; Jung et al., 2012; Shigeoka et al., 2016), so as to normalize the suppressed neural development in the *Tbr1*^K228E/K228E^ brain.

These GSEA results collectively suggest first that heterozygous and homozygous TBR1-K228E mutations in mice at E16.5 lead to both shared and distinct changes in transcriptomic profiles. Perhaps a more interesting observation is that the heterozygous TBR1-K228E mutation leads to ASD-related transcriptomic patterns that mimic those observed in ASD, whereas the homozygous TBR1-K228E mutation leads to transcriptomic changes opposite to those observed in ASD (Figure 4C). This highlights the importance of studying heterozygous *Tbr1*-mutant mice in exploring ASD-relevant pathophysiological mechanisms. In this context, the synapse-related gene sets that were negatively enriched in the *Tbr1*^+/K228E^ (but not *Tbr1*^K228E/K228E^) transcriptome might contribute to the development of ASD-related phenotypes, such as abnormal neuronal projections and development of neural circuits. These points, however, do not lessen the importance of the transcriptomic phenotypes shared by *Tbr1*^K228E/K228E^ and *Tbr1*^+/K228E^ mice, such as astrocyte- and ribosome-related genes, which may be more directly associated with the role of TBR1 in the regulation of embryonic and cortical development.

One of the most unexpected results in the current study was the reciprocal change in the density of Pv-positive interneurons in superficial and deep cortical layers in the prelimbic region of the mPFC in heterozygous *Tbr1^+/K228E^* mice (Figure 5)—specifically, increased density in deep layers and decreased density in superficial layers. These changes were not associated with similar changes in the density of SST- or VIP-positive interneurons. It has been shown that *Tbr1* is primarily expressed in excitatory glutamatergic neurons in various brain regions, including the neocortex (Hevner et al., 2001, 2003). Therefore, the altered densities of Pv interneurons might be associated with certain primary changes occurring in neocortical glutamatergic neurons. For instance, decreased activity of layer 6 pyramidal neurons in *Tbr1*^+/K228E^ mice might suppress the projection of layer 6 cortical pyramidal neurons to the thalamus, as reported previously (Hevner et al., 2001, 2002), which would subsequently suppress the thalamocortical pathways that project to neocortical pyramidal neurons and interneurons (Delevich et al., 2015), pathways that are known to be important for the tangential migration of interneurons from deep to superficial cortical layers in the developing cortex (Tuncdemir et al., 2016).

Results of electrophysiological analyses of layer 6 pyramidal neurons (Figure 6) are in line with the increased density of Pv interneurons in deep cortical layers. Specifically, the frequency, but not amplitude, of mIPSCs was increased in layer 6 pyramidal neurons in *Tbr1^+/K228E^* mice at 2–3 months age, the developmental stage at which Pv interneuronal densities were quantified. This result might indicate that, because of their increased number, Pv interneurons may provide strong inhibitory synaptic input onto target layer 6 pyramidal neurons. Notably, the extent of the increases in mIPSC and sIPSC frequencies is greater than that in the number of Pv interneurons in *Tbr1*^+/K228E^ mice, suggesting the possibility that the increased mIPSC and sIPSC frequencies might involve increases in inhibitory synapse formation, presynaptic release at inhibitory synapses, or the excitability of Pv interneurons. Intriguingly, network activity increased excitatory synaptic input onto these pyramidal neurons, an effect that likely serves to normalize the synaptic E/I balance. Whether this compensation normalizes the output function of layer 6 pyramidal neurons in brain slices or *in vivo* remains to be determined. Pv interneurons have also been implicated in the regulation of brain oscillations in the gamma range and cognitive brain functions (Cardin et al., 2009; Sohal et al., 2009; Cardin, 2016). Therefore, the increased mIPSC and sEPSC frequencies in the *Tbr1*^+/K228E^ mPFC might disrupt the normal functions of Pv interneurons.

*Tbr1^+/K228E^* mice showed abnormal behaviors in social, repetitive behavioral, anxiety, and locomotor domains. Specifically, these mice showed ASD-like impaired direct social interaction and increased repetitive behavior, including self-grooming (Figure 7). In addition, *Tbr1*^+/K228E^ mice showed increased anxiety-like behavior (elevated plus-maze and light-dark tests) and modestly increased locomotor activity (Laboras test; Figure 8). Determining how the heterozygous TBR1-K228E mutation leads to these behavioral abnormalities at the circuit level may take extensive additional investigations. However, a previous study reported that social deficits can be induced in WT mice by optogenetically stimulating mPFC pyramidal neurons and that the resulting social deficits can be rescued by activation of Pv interneurons (Yizhar et al., 2011). In addition, *Cntnap2*-knockout mice, a mouse model of ASD (Peñagarikano et al., 2011), display pyramidal neuronal hyperactivity and social deficits that are normalized by stimulation of Pv interneurons (Selimbeyoglu et al., 2017). Moreover, mice harboring a neuroligin 3-R351C mutation, another mouse model of ASD (Tabuchi et al., 2007), display abnormal gamma oscillations involving Pv interneuronal hypo-excitability and behavioral deficits that are normalized by Pv interneuronal stimulation at a gamma frequency (40 Hz) nested at a theta frequency (8 Hz; Cao et al., 2018). Although these studies largely implicate decreased functions of Pv interneurons in the regulation of local brain oscillation and social interaction, the converse situation involving increased Pv neuronal density and output, as in our current study, might also induce functional abnormalities of Pv interneuron networks in the mPFC.

*TBR1* has been strongly linked to ASD (Neale et al., 2012; O’Roak et al., 2012, 2014; Traylor et al., 2012; Abrahams et al., 2013; De Rubeis et al., 2014; Deriziotis et al., 2014; Hamdan et al., 2014; Palumbo et al., 2014; Chuang et al., 2015; Bowling et al., 2017; Geisheker et al., 2017; McDermott et al., 2018; Vegas et al., 2018). Perhaps one of the most important results of our study is that a heterozygous TBR1 point mutation (TBR1-K228E) from a human ASD patient can actually induce ASD-like behavioral phenotypes in mice, establishing a causal relationship and thus *prima facie* evidence of validity. In addition, our study provides relevant mechanisms at transcriptomic, synaptic, and cell biological levels. Specifically, DEG and GSEA analyses identified genes or gene sets that have been strongly associated with ASD, including high-risk ASD genes (Abrahams et al., 2013), genes involved in brain development (Brambilla et al., 2003; Courchesne et al., 2007; Walsh et al., 2008; Hazlett et al., 2017), and genes associated with neuronal synapses (Zoghbi, 2003; Garber, 2007; Südhof, 2008; Bourgeron, 2009; Spooren et al., 2012; Jiang and Ehlers, 2013; Won et al., 2013; Ebrahimi-Fakhari and Sahin, 2015; Monteiro and Feng, 2017) and astrocytes (Clarke and Barres, 2013; Petrelli et al., 2016). In addition, our study demonstrated that Pv interneurons, which have been strongly implicated in ASD (Lawrence et al., 2010; Yizhar et al., 2011; Saunders et al., 2013; Barnes et al., 2015; Filice et al., 2016; Selimbeyoglu et al., 2017; Cao et al., 2018; Hashemi et al., 2018; Lee et al., 2018), exhibit an altered density in *Tbr1*^+/K228E^ mice. Moreover, the increased inhibitory synaptic transmission in layer 6 pyramidal neurons supports the role of an E/I imbalance in ASD (Rubenstein and Merzenich, 2003; Pizzarelli and Cherubini, 2011; Nelson and Valakh, 2015; Lee et al., 2017).

Lastly, a recent study on mice with a deletion in *Tbr1* restricted to cortical layer 6 starting from late gestation was shown to display various ASD-related phenotypes (Fazel Darbandi et al., 2018). An RNA-Seq analysis of these mice showed altered expression of many genes associated with brain development and ASD risk (Fazel Darbandi et al., 2018), similar to our results. In addition, these mice showed decreases in the number of both excitatory and inhibitory synapses and decreased neuronal excitability, as supported by increased hyperpolarization-activated cation currents in layer 6 pyramidal neurons in the somatosensory cortex. These synaptic changes contrast with the synaptic and neuronal changes observed in our *Tbr1*^+/K228E^ mice: increased mIPSC frequency and sEPSC frequency, but normal excitability of layer 6 pyramidal neurons. This discrepancy might be attributable to the use of layer 6 pyramidal neurons from different cortical areas (mPFC vs. somatosensory), deletion of the gene in different brain regions and cell types (whole-brain vs. layer 6 pyramidal neurons), and/or introduction of different types of mutations (a point mutation that increases protein level vs. an exon-deleting mutation that decreases protein level) in the two studies.

In summary, our study demonstrates that the heterozygous TBR1-K228E mutation identified in individuals with ASD can induce ASD-like behavioral phenotypes in mice. In addition, our study reveals various abnormalities at transcriptomic, protein, cellular, and synaptic levels that may underlie the behavioral deficits observed in *Tbr1^+/K228E^* mice.

## Data Availability Statement

The datasets generated for this study can be found in the Gene Expression Omnibus (GEO) under accession numbers GSE134526, GSM3955156, GSM3955157, GSM3955158, GSM3955159, GSM3955160, GSM3955161, GSM3955162, GSM3955163, GSM3955164, GSM3955165, GSM3955166, GSM3955167, GSM3955168, GSM3955169, GSM3955170.

## Ethics Statement

The animal study was reviewed and approved by Committee of Animal Research at KAIST.

## Author Contributions

S-GK generated Tbr1 mice. DK performed TBR1-K228E biochemical assays. S-GK performed immunoblot experiments. SK performed behavioral experiments. CY and KK performed electrophysiological experiments. CY performed immunohistochemistry. SK and HK performed RNA-Seq analysis. SK and EK designed research and wrote the manuscript.

## Conflict of Interest

The authors declare that the research was conducted in the absence of any commercial or financial relationships that could be construed as a potential conflict of interest.

## Footnotes

^1^http://www.informatics.jax.org/homology.shtml

^2^http://software.broadinstitute.org/gsea

^3^http://software.broadinstitute.org/gsea/msigdb

^4^http://software.broadinstitute.org/gsea/

## Supplementary Material

FIGURE S1 qRT-PCR analyses for some of the DEGs from *Tbr1^+/K228E^* and *Tbr1*^K*228E /K228E*^ mice. **(A)** qRT-PCR analyses were performed for all five DEGs from *Tbr1*^+/K228E^ mice [(E16.5; LMBRD1, LYPD6, PDE3B, ILK, and CRX) and six DEGs (Tbr1 and six DEGs that are associated with ECM and extracellular GO terms indicated by underline (Figures 3B,C)] from *Tbr1*^ K*228E /K228E*^ mice (E16.5). The genes that are increased and decreased in the expression in RNA-Seq analyses are indicated by red and blue colors, respectively. *n* = 5 mice (males and females mixed) for WT, *Tbr1*^+/K228E^, and *Tbr1*^+/K228E^, **P* < 0.05, ****P* < 0.001, one-way ANOVA with Dunnett’s test.

FIGURE S2 Ctip2 marks layer 5 more strongly than layer 6. **(A)** Ctip2 marks layer 5 more strongly than layer 6 (lower two images) and, together with Tbr1, reveals the boundary between layers 5 and 6 (upper two images). The upper two images were borrowed from Figure 5A for comparison.

TABLE S1 All RNA-Seq data.

TABLE S2 Statistical results.
