# Supplementary figures and images for: A TBR1-K228E Mutation Induces Tbr1 Upregulation, Altered Cortical Distribution of Interneurons, Increased Inhibitory Synaptic Transmission, and Autistic-Like Behavioral Deficits in Mice
Source: Front Mol Neurosci. 2019 Oct 9;12:241. doi: 10.3389/fnmol.2019.00241 (PMC6797848; doi:10.3389/fnmol.2019.00241)

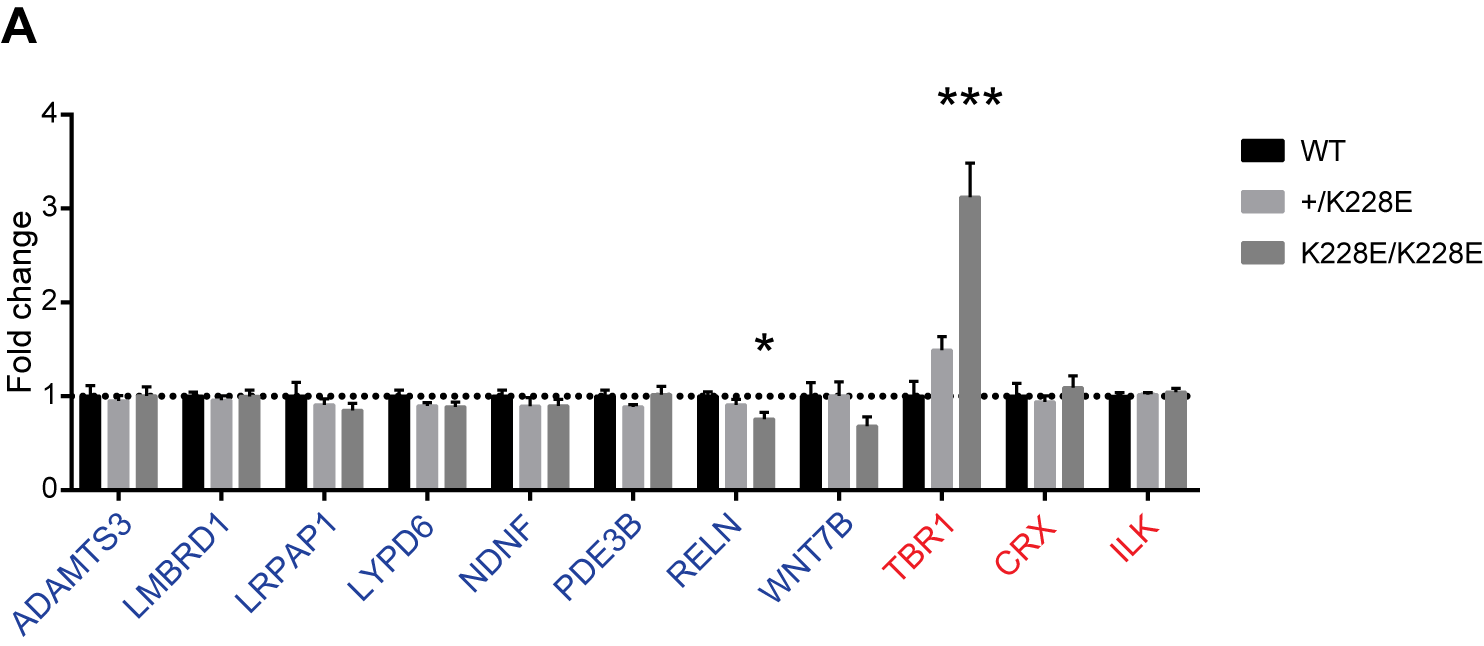

Supplement: FIGURE S1 — qRT-PCR analyses for some of the DEGs from Tbr1+/K228E and Tbr1K228E /K228E mice. (A) qRT-PCR analyses were performed for all five DEGs from Tbr1+/K228E mice [(E16.5; LMBRD1, LYPD6, PDE3B, ILK, and CRX) and six DEGs (Tbr1 and six DEGs that are associated with ECM and extracellular GO terms indicated by underline (Figures 3B,C)] from Tbr1 K228E /K228E mice (E16.5). The genes that are increased and decreased in the expression in RNA-Seq analyses are indicated by red and blue colors, respectively. n = 5 mice (males and females mixed) for WT, Tbr1+/K228E, and Tbr1+/K228E, *P < 0.05, ***P < 0.001, one-way ANOVA with Dunnett’s test. [file Image_1.TIF]

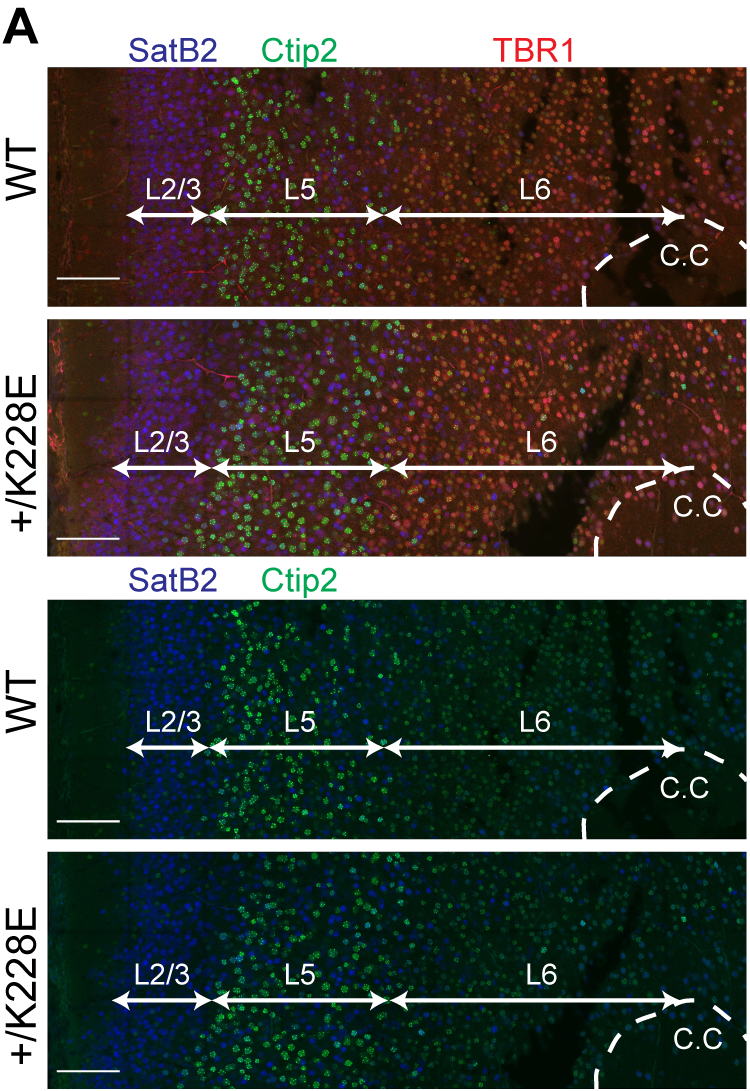

Supplement: FIGURE S2 — Ctip2 marks layer 5 more strongly than layer 6. (A) Ctip2 marks layer 5 more strongly than layer 6 (lower two images) and, together with Tbr1, reveals the boundary between layers 5 and 6 (upper two images). The upper two images were borrowed from Figure 5A for comparison. [file Image_2.TIF]
